# TSPO imaging in animal models of brain diseases

**DOI:** 10.1007/s00259-021-05379-z

**Published:** 2021-07-10

**Authors:** Nadja Van Camp, Sonia Lavisse, Pauline Roost, Francesco Gubinelli, Ansel Hillmer, Hervé Boutin

**Affiliations:** 1 Université Paris-Saclay, CEA, CNRS, MIRCen, Laboratoire des Maladies Neurodégénératives, 92265 Fontenay-aux-Roses, France; 2 Department of Psychiatry, Yale School of Medicine, New Haven, CT USA; 3 Department of Radiology & Biomedical Imaging, Yale School of Medicine, New Haven, CT USA; 4 Department of Biomedical Engineering, Yale School of Engineering & Applied Science, New Haven, CT USA; 5 Division of Neuroscience and Experimental Psychology, School of Biological Sciences, Faculty of Brain and Mental Health, University of Manchester, M13 9PL, Manchester, UK; 6 Wolfson Molecular Imaging Centre, University of Manchester, 27 Palatine Road, M20 3LJ Manchester, UK; 7 Geoffrey Jefferson Brain Research Centre, Manchester Academic Health Science Centre, Northern Care Alliance & University of Manchester, Manchester, UK

**Keywords:** Translocator protein 18 kDa, Neuroinflammation, Alzheimer’s disease, Parkinson’s disease, Stroke, Multiple sclerosis, Positron emission tomography

## Abstract

Over the last 30 years, the 18-kDa TSPO protein has been considered as the PET imaging biomarker of reference to measure increased neuroinflammation. Generally assumed to image activated microglia, TSPO has also been detected in endothelial cells and activated astrocytes. Here, we provide an exhaustive overview of the recent literature on the TSPO-PET imaging (i) in the search and development of new TSPO tracers and (ii) in the understanding of acute and chronic neuroinflammation in animal models of neurological disorders. Generally, studies testing new TSPO radiotracers against the prototypic [^11^C]-R-PK11195 or more recent competitors use models of acute focal neuroinflammation (e.g. stroke or lipopolysaccharide injection). These studies have led to the development of over 60 new tracers during the last 15 years. These studies highlighted that interpretation of TSPO-PET is easier in acute models of focal lesions, whereas in chronic models with lower or diffuse microglial activation, such as models of Alzheimer’s disease or Parkinson’s disease, TSPO quantification for detection of neuroinflammation is more challenging, mirroring what is observed in clinic. Moreover, technical limitations of preclinical scanners provide a drawback when studying modest neuroinflammation in small brains (e.g. in mice). Overall, this review underlines the value of TSPO imaging to study the time course or response to treatment of neuroinflammation in acute or chronic models of diseases. As such, TSPO remains the gold standard biomarker reference for neuroinflammation, waiting for new radioligands for other, more specific targets for neuroinflammatory processes and/or immune cells to emerge.

## General introduction

Nowadays, the 18-kDa translocator protein (18-kDa TSPO) is the most prominent biomarker for imaging of glial activation and has been reviewed more than a hundred times over the last two decades (cf. PubMed search terms: “(omega* OR TSPO OR PBR OR PK11195) AND (PET OR positron) AND (brain OR cereb*) AND (English[ lang])” and selecting “review” or “systematic review”). About 40 years ago, this protein was named *peripheral benzodiazepine receptors* (or omega-3 binding site) due to its pharmacology (high affinity for some benzodiazepines) and its preferential expression in the myocardium, kidney and adrenals [1–3]. Its overexpression in brain lesions rapidly identified this protein as a potential in vivo imaging marker of inflammation-related neurodegeneration, notably in models of brain ischaemia [4–8]. One of the first applications of TSPO/PBR radioligands for positron emission tomography (PET) imaging was to image brain tumours in man [9, 10]. It was only in the mid- to late 1990s, using animal models of brain diseases that an increased level of TSPO/PBR was proven to be associated with microglial activation [11–14]. Preclinical models are of great utility when it comes to testing, validating and implementing new biomarkers, imaging methods and contrast agents. The preclinical contribution in the development of new radioligands for TSPO here is no exception, as underlined by its representation in the literature – more than a third of the abovementioned reviews.

Our aim here is to provide an exhaustive overview of the recent literature on the use of animal models in two major applications in the field of preclinical TSPO-PET imaging: either as a tool to develop new tracers and/or imaging methodologies or as a model to understand the contribution of acute and chronic neuroinflammation in neurological disorder models.

## Models of acute neuroinflammation

### Tracer development

The animal models of acute neuroinflammation described below have mostly been used for the development and validation of TSPO tracers as they provide generally a consistent, robust and focal neuroinflammatory response and TSPO expression with a well-described time course. From a tracer development perspective, on top of classical brain tracer characteristics such as high blood-brain barrier penetrance, neuroinflammation and TSPO tracers have specific highly desirable characteristics that are summarized in Table 1.

**Table 1:** Required characteristics for new neuroinflammation and TSPO tracers

Challenges for new neuroinflammation tracers	Specific requirements for new TSPO tracers
High metabolic stability	
High free ligand availability in plasma (i.e., low binding to circulating cells or proteins)	
High brain availability (i.e., high blood-brain barrier penetrance, not *P-gp* substrate)	
N/A	No sensitivity to the TSPO rs6971 polymorphism
Development of cell-specific ligands: microglia vs astrocytes	N/A
Neuroinflammatory phenotypic selectivity: pro- vs anti-inflammatory	N/A

*P-gp*, P-glycoprotein; *N/A*, not applicable

### Lipopolysaccharide (LPS) model

LPS, also referred to as endotoxin, is a major component of the outer membrane of gram-negative bacteria, with lipid A in the membrane and the O antigen constituting the outer-facing surface of the bacterium. Soluble endotoxin is released when bacteria are destroyed but is also released physiologically as outer membrane vesicles [15]. LPS is a potent inducer of inflammation both in peripheral tissues and in the central nervous system via the activation of toll-like receptor 4 (TLR4), which results in NF-κB transcriptional activation of hundreds of inflammatory genes, including pro-inflammatory cytokines such as TNFα, IL-6 and pro-IL-1β. LPS administration in preclinical models is performed systematically (intravenous, i.v., or intraperitoneal, i.p.) or by stereotactic intracerebral injection. In this topic, we will discuss TSPO-PET imaging studies in models using both administration routes. We used the following search in PubMed, “(omega* OR TSPO OR PBR OR PK11195) AND (PET OR positron) AND (brain OR cereb*) AND (LPS OR lipopolysaccharide) AND (rat OR mouse OR mice OR animal* OR experimental OR pig OR gerbil OR rabbit OR guinea OR primate OR dog OR cat) AND (English[ lang])”, which returned initially 27 records. After exclusion of those that were not PET imaging studies that used LPS as a comorbidity factor and those not using LPS as a model per se, 18 references were retained to TSPO-PET imaging in LPS models.

It has been a matter of discussion how peripherally administered LPS induces neuroinflammation in the central nervous system. Initially, it was suggested that LPS-induced neuroinflammation was an indirect effect through TNFα, which crosses the blood-brain barrier [16]. However, it has been shown more recently that LPS infiltrates the brain via lipoprotein receptor (such as SR-B1) transport in which LPS binds specifically to those receptors at the blood-brain interfaces [17]. These receptors are located in *astrocytes* (as part of immunologic functional barriers), tanycytes that are in direct contact with the cerebrospinal fluid along the ventricular walls and centrum ventricular organs) and *endothelial cells* (arbitrating a neuroimmune communication by actively responding to immune challenges such as LPS through the release of cytokines and other mechanisms) [17]. Cytokine production and direct interaction of LPS with TLR4 induce microglial activation, the first-line defence of the brain. Using adult mice receiving a single intraperitoneal administration of LPS (1 mg/kg), it has been demonstrated by in vitro immunohistochemical studies that microglial proliferation occurs nonuniformly in the central nervous system but in a region- and dose-dependent manner. This is followed by a return to control levels 20 days after a single stimulation independent of the LPS dose (100 μg/kg or 1 mg/kg LPS) [18]*.* Altogether, various studies confirm that acute systemic LPS administration induces transient inflammation in the central nervous system [19, 20, 15], which makes LPS an appealing approach to measure the “neuroimmune response” by imaging TSPO levels. Moreover, at a low dose, the pro-inflammatory properties of endotoxin mimic the complex responses to systemic infection. However, due to the transient effect of acute endotoxin, consistent timing of imaging is required to ensure consistent results. Ota et al. were the first to image the neuroinflammatory response after a systematic LPS administration in the rat brain (2mg/kg, i.p.) using [^11^C]-R-PK11195 [21]. Animals were scanned before and 2 days after LPS administration, and the difference in SUV uptake was estimated with voxel-wise statistical parametric mapping. This revealed a striato-cortical cluster, which surprisingly reflected a significant decrease of the TSPO-PET signal rather than an increase. As this result was not further supported by any ex vivo or in vitro data, additionally considering both the limitations of the SUV measure in the absence of any blood data and the low specificity of [^11^C]-R-PK11195, interpretation of these data needs to be considered cautiously. A far more complete study in the rat brain was performed by Schmidt et al., who evaluated the time and regional course of the [^11^C]PBR28 TSPO-PET signal after a systemic (i.p.) LPS challenge (3 mg/kg) in the context of a genetic risk factor for Parkinson’s disease (more details in the paragraph “Parkinson’s disease” below) [22]. This study reported a reliable and consistent neuroinflammatory response 24 h after the challenge as visualized by in vitro autoradiography using [^11^C]PBR28. Subsequently, a longitudinal in vivo [^11^C]PBR28 PET study demonstrated a significant detectable LPS-induced TSPO-PET response 10 months after administration, particularly in the cortical and ventral regions. As such, TSPO response to peripheral inflammation can also be monitored in an ethically viable manner by minimizing pathogenic risk to human subjects, providing an important model for studying immune signalling in human research [23]. TSPO imaging can leverage this model by conducting scans before and after endotoxin challenge, to measure the change in TSPO brain levels. The results provide important information on the brain’s immune response to a stimulus, or immune function, to complement the snapshots of the brain’s neuroimmune state assessed by single baseline TSPO scans. Such endotoxin protocol was first applied to non-human primates (baboons), where serial [^11^C]PBR28 images were acquired after i.v. administration of 0.1-mg/kg endotoxin [24]. This dose increased the [^11^C]PBR28 volume of distribution (*V*_T_) by +29% and +62% relative to baseline 1 h and 4 h after endotoxin injection, respectively. These results demonstrated the time dependence of measurements of endotoxin effects. The peak response occurred 3–5 h post-dose, which informed the design for future human studies. In humans, acute endotoxin administration (1.0 ng/kg LPS, i.v.) induced a 30–60% increase in [^11^C]PBR28 *V*_T_ in the brain 3 h after injection [25]. In the rhesus monkeys, a similar dose (1.0 ng/kg LPS, i.v.) increased [^11^C]PBR28 *V*_T_ by +39% from baseline 2.5 h post-endotoxin [26].

Altogether, a comparison across different TSPO-PET studies after the LPS challenge highlights an important species difference in the LPS sensitivity. Globally 2.5 to 4 h, a comparable TSPO response (+40 to +60% increase) after LPS challenge could be observed in baboon, rhesus NHP, and human, but the LPS dose administered in baboon was 10^5^-fold higher compared to the doses in rhesus or human. Importantly, both the baboon and human studies demonstrated that TSPO-PET brain changes after endotoxin challenge were accompanied with changes in subjective sickness behaviour and blood levels of inflammatory cytokines. In the rodent, even higher doses (in the order of mg/kg) are needed to evoke a detectable neuroinflammatory TSPO response (of note, the administration route in rodents is i.p. vs i.v. in NHP and humans). Altogether, this suggests that rhesus monkeys and humans exhibit greater sensitivity to endotoxin compared to rodents and baboons.

The neuroinflammatory response to systemic endotoxin depends on more factors. A study on mice systematically treated with LPS (0.63 mg/kg) revealed significant age and sex influences, which were reflected by both differential expression of pro-inflammatory factors and an increased ex vivo TSPO ligand uptake, using [^18^F]VC701, in aged female mice [27]. Furthermore, the brain regions displayed differential susceptibility to LPS, which is attributable to the number of microglia present and the levels of inflammation-related factors produced by these cells [28]. As such, the systemic endotoxin model can provide valuable new insight on the role of inflammation in the pathogenesis of brain diseases such as stroke, and Parkinson’s disease, which will be discussed in the next chapters. LPS-induced systemic inflammation was further used to evaluate the influence on the [^11^C]-R-PK11195 TSPO-PET signal in the presence of a pre-existing neuroinflammatory lesion [29]. Here, the authors suggested that the TSPO-PET signal refers to the level of microglial activation rather than the number in activated microglia, an observation that was contradicted by Tournier et al. as discussed below [30]. Finally, Vignal et al. [31] used TSPO-PET imaging to evaluate the impact of immune regulating factors on brain [^18^F]FEPPA TSPO-PET response. An overview of the studies is presented in Table 2.

**Table 2:** Overview of TSPO imaging studies in different LPS models

PET/SPECT tracer	Rational	LPS model	Species	Imaging time point	Major outcomes and additional readouts	Ref.
[^18^F]FEPPA	Optimization of radiosynthesis; impact of astroglial connexin 43 (Cx43) on brain immunoregulation	Systemic administration (5 mg/kg, i.p.) Cx43^fl/fl^/hGFAP-Cre; Cx43^fl/fl^ model	C57Bl/6J mice	24 h post-injection	• Systemic inflammation results in two-fold increase in TSPO-PET. • Deletion of astrocyte Cx43 abolishes the LPS-induced TSPO increase	[45, 31]
[^11^C]-R-PK11195	Impact of systemic inflammation on toxic-induced neuroinflammation	Systemic administration (10 mg/kg, i.p.) after intracerebral ethanol-induced neuroinflammation	Rats	4 days post-injection	LPS induced higher TSPO-PET signal. The number of activated microglia in the neurotoxic lesion is similar in both conditions. LPS induced higher expression of inflammatory cytokines	[29]
[^18^F]VC701	Evaluation of age and sex differences	Systemic administration (0.63 mg/kg)	C57Bl/6J mice	6 h post-injection	Pro-inflammatory response in aged female is higher compared to adult female and aged male	[27]
[^11^C]PBR28	To evaluate the impact of systemic inflammation on TSPO-PET in the brain; development of a paradigm to measure dynamic microglial changes in the NHP brain	Systemic administration: 0.1 mg/kg, i.v., and 1 ng/kg, i.v.	NHP: baboon Rhesus monkey	1–5.5 h and ∼22 h post-injection 2.5 h, 14 days post-injection	LPS-induced systemic inflammation produces a detectable TSPO-PET signal in the brain mediated by inflammatory cytokines. The colony-stimulating factor 1 receptor kinase inhibitor depletes brain microglia and induces a significant decrease in the TSPO-PET signal, which recovers after 12 days	[26, 24]
[^11^C]PBR28	To evaluate the impact of systemic inflammation on TSPO-PET in the brain	Systemic administration (1 ng/kg, i.v.)	Human	3h	First in a human demonstration that a systemic LPS challenge induces microglial activation in the brain	[25, 46]
[^11^C]-R-PK11195	Model of maternal infection as a risk factor for periventricular leukomalacia and cerebral palsy (CP) in neonates	Intra-uterine exposure in pregnant rabbits (20 μg/kg)	Rabbit	1–7 days post-injection	Increased TSPO-PET in the brain and increased inflammatory markers are detected up to 2 weeks after birth	[47]
[^18^F]DPA-713	To evaluate if TSPO-TEP reveals specifically the pro- or anti-inflammatory phenotype	Cultured rodent astrocytes, microglia and macrophages stimulated with TNF, LPS and IL-4; mice injected with AdTNF or IL-4	Mice		TSPO expression corresponds to a pro-inflammatory phenotype	[33]
[^123^I]CLINDE	Determine the cell population in which TSPO is altered using fluorescence-activated cell sorting on radioligand-treated tissue	Intracerebral (hippocampus) (10 μg)	Rat	3 days post-injection	LPS induces a microglial expansion and an increase in microglial TSPO binding	[30]
[^11^C]-R-PK11195, [^11^C]DAA1106, [^11^C]PBR28, [^18^F]DPA-714, [^18^F]GE-180, [^18^F]fluoromethyl-PBR28	Validation and comparison of TSPO-PET ligands; validation of pharmacokinetic quantification models	Intracerebral (striatum) (1 μg, 10 μg, 50 μg)	Rat	3–4 days post-injection/longitudinal after injection	2nd generation TSPO ligands reveal a higher uptake compared to that of [^11^C]-R-PK11195. IHC characterization of the LPS model	[40, 35, 38, 44, 39, 48–51, 34, 52]
[^18^F]CB251	Comparison and validation of a new 3rd generation TSPO ligand; characterization and identification of immune cells contributing to LPS-induced neuroinflammation	Intracerebral (striatum) (5 μg/2 μl)	Mice	4 days post-injection	[^18^F]CB251 does not show a differential affinity for the TSPO polymorphism; following LPS-induced neuroinflammation, peripheral immune cells cross the BBB and actively contribute to the TSPO-PET signal. Bioluminescence studies to identify the contribution of peripheral immune cells to the brain immune response	[37]

Intracerebral administered LPS *directly* interacts with TLR4 located on astrocytes and microglia, hence locally activating pro-inflammatory microglia and astrocytes. Neurotoxic reactive astrocytes can also be activated *indirectly* through pro-inflammatory factors expressed by microglia [32]. Even though both pro-inflammatory microglia and astrocytes contribute to the LPS-induced TSPO-PET signal [33–35, 30], microglia are abundantly present over astrocytes, suggesting that the TSPO-PET signal in the intracerebral induced LPS model is mainly due to an increase in microglial density. In vitro studies demonstrated that LPS slightly decreased TSPO gene expression in human microglia and [^3^H]PBR28 binding sites in human macrophages [36]. Building on these findings, an innovative ex vivo technique using fluorescence-activated cell sorting of radioligand-treated tissue reported that while LPS did not change radioactivity per cell in microglia, LPS dramatically increased the number of microglia per gram of tissue [30]. Despite possible LPS binding to endothelial cells [17], there is no evidence for changes in TSPO levels in endothelial cells following LPS stimulation [33]. Based on the robust imaging [25] and immunohistochemistry [34] literature reporting LPS-induced increases in TSPO signal, findings point to LPS increasing the number of TSPO-expressing microglia and not the number of TSPO per microglia. TSPO imaging of acute LPS effects should be more accurately characterized as capturing “microglial proliferation”, rather than assessing “microglial activation”. A recent multimodal imaging study combining TSPO-PET and bioluminescence imaging demonstrated that peripheral immune cells cross the blood-brain barrier, exacerbate the LPS-induced neuroinflammation to produce higher levels of neurotoxicity and actively contribute to the TSPO-PET signal. For this study, the authors used a new TSPO ligand, [^18^F]CB251, which has a higher affinity for TSPO compared to ligands such as PK11195, PBR28 and GE-180 [37]. In general, intracerebral induced neuroinflammation by LPS is an attractive experimental model to confirm or compare the sensitivity of new TSPO radioligands in a situation of acute immune activation in rodents [34, 38–40]. Additionally, this model is also used to evaluate new PET ligands specific for other pro-inflammatory microglial-specific targets, such as CB2 and P2X7, in comparison to validated TSPO ligands [35, 41]. A review of the literature highlights the important differences between experimental protocols in terms of injected dose, site of injection and the time point of imaging (Table 2). Intracerebral LPS doses vary from 1 [35, 40] to 5–10 μg [42, 43] and even up to 50 μg [34, 44]; injection is generally performed in the striatum but also in the hippocampus and substantia nigra; finally, imaging time points vary from hours to days or even weeks post-injection [42, 43]. These experimental differences undoubtedly affect the neuroinflammatory response and its imaging readout, rendering the comparison between studies almost impossible.

### Stroke

Neuroinflammation was truly identified as an essential component of the pathophysiology of stroke in the mid- to late 1990s [53–55], although the identification of TSPO glial expression was originally described after cerebral ischaemia, when TSPO was still referred to as the peripheral benzodiazepine receptor [56, 7]. Only about 10–15 years later, those ex vivo observations in animal models were confirmed by in vivo PET imaging in clinical settings [57–59]. In 2015, Boutin and Pinborg published a review on the literature of TSPO-PET imaging in stroke covering brain ischaemia models and clinical studies [60]. Overall, that review [60] covered clinical studies investigating (i) TSPO after stroke using [^11^C]-R-PK11195 [58], (ii) preclinical studies in primates [61, 62] using a clinical scanner and [^11^C]-R-PK11195 as well as (iii) the first preclinical studies using small animal dedicated scanners. These studies aimed to evaluate – at the time – new tracers such as [^11^C]PBR28 [63] or [^18^F]DPA-714 [64] or better determine the time course of TSPO expression following focal ischemia [65]. Altogether, these first studies were extremely helpful to demonstrate the importance of neuroinflammation after stroke and the value of TSPO-PET imaging to quantify it, and they provided invaluable information in terms of the study design to future preclinical and clinical studies. Therefore, in the following paragraphs, we will only review the literature on TSPO imaging in stroke models that has been published since the most recent paper included in that review at the time of publication, which was Tiwari et al. [66]. For this section, we used the following search in PubMed, “(omega* OR TSPO OR PBR OR PK11195) AND (PET OR positron) AND (brain OR cereb*) AND (stroke OR ischem* OR ischaem*) AND (rat OR mouse OR mice OR animal* OR experimental OR pig OR gerbil OR rabbit OR guinea OR primate OR dog OR cat) AND (English[ lang])”, limiting the search to 2015–2021. This returned initially 52 records, excluding those not using stroke models, reviews and clinical research papers, and 26 references were finally kept for this section. The results of all these studies have been summarized in Table 3; most of them did confirm their in vivo PET results using immunohistochemistry and/or autoradiography, but these results will not be discussed in full details here; instead, a short summary of those ex vivo/in vitro results can be found in Table 3.

**Table 3:** Overview of the preclinical TSPO-PET studies in experimental stroke models (published since 2015 [60]).

PET tracer *	Rational	Stroke model and species	Imaging time points	Main imaging findings	Additional readouts	Ref.
[^18^F]GE-180 [^11^C]-R-PK11195	Validation of [^18^F]GE-180 as new TSPO tracer by direct comparison with [^11^C]-R-PK11195	60-min intraluminal MCAO in male Wistar rats (357±44g)	5–6 days post-MCAO	• **Better signal-to-noise ratio with [**^**18**^**F]GE-180; BP**_**ND**_**=3.5±0.4 for [**^**18**^**F]GE-180 > BPND=2.4±0.5 for [**^**11**^**C]-R-PK11195** • **Percentage intact [**^**18**^**F]GE-180 in brain: 98±2%, 96±3% and 94±2% at 10, 30 and 60 min post-injection**	PET and ARG: displacement by unlabelled R-PK11195 or GE-180) confirmed TSPO specificity and in vivo results	[67]
[^11^C]-R-PK11195 2[^18^F]-fluoro-A85380 (nAChR α4β2) [102]	Investigate the role of nAChR α4β2 in neuroinflammation; TSPO [^11^C]-R-PK11195 imaging used as reference biomarker/tracer	2-h intraluminal MCAO in male SD rats (300 g)	1, 3, 7, 14, 21, and 28 days post-MCAO	• **Increase in** 2[^18^F]-fluoro-A85380 and **[**^**11**^**C]-R-PK11195 uptake at days 3–7 post-MCAO** • **Decrease of both tracer uptake at 14–28 days** • **nAChR α4β2 antagonist increased [**^**11**^**C]-R-PK11195 uptake**	PET results confirmed by IHC for nAChR α4β2 and TSPO	[96]
[^18^F]FEBMP	Evaluation of [^18^F]FEBMP as new TSPO tracer	30-min intraluminal MCAO in 8–9-weeks-old (240–330 g) male SD rats	7 days post-MCAO	• **Ipsi-/contralateral ratio: 3.20±0.12 (BPND: 2.72±0.27) in the infarcted striatum** • **Percentage intact [**^**18**^**F]FEBMP in the brain: 83.2±7.4% and 76.4 ± 2.1% at 30 and 60 min post-injection**	Human brain tissue ARG: no sensitivity of [^18^F]FEBMP to TSPO rs6971 polymorphism (comparison with [^11^C]-R-PK11195, [^11^C]PBR28, [^11^C]AC-5216, and [^11^C]DAA1106)	[66]
[^11^C]-R-PK11195 (USPIO for detection of phagocytic cells)	Mapping of activated microglia *vs* phagocytic cells	Permanent microsphere-induced MCAO in male Wistar rats (320–365 g)	PET at day 6, 27 and 55 USPIO MR at day 7, 27, 56	• USPIO+ only tissue at day 7 progresses to a necrotic cavity at days 28–56 • **USPIO+ and TSPO+ or TSPO+-only tissue remains viable at days 28–56** • **USPIO+ and TSPO+ signal is detected at days 28–56 in the thalamus which was not in the initial infarct core**	PET and MR data confirmed by histological and IHC staining	[85]
[^15^O]H_2_O (CBF) [^11^C]-R-PK11195 [^18^F]FDG	Investigate the contribution of NI to [^18^F]FDG signal	Permanent microsphere-induced MCAO in male Wistar rats (320–363 g)	7 days pre-MCAo and at days 2, 7, 14, 21 and 42 post-MCAO	• NI occurs principally in the ischemic brain region, i.e. without sufficient metabolic supply • NI may mask even more severe hypometabolism as NI itself increases [^18^F]FDG uptake	PET and T2 MR data confirmed by histology and IHC	[87]
[^11^C]-R-PK11195 [^18^F]FLT (NSC proliferation)	Investigate the effects of tDCS on NI and NSC proliferation	60-min intraluminal MCAO in male Wistar rats (290–330 g)	[^18^F]FLT and [^11^C]-R-PK11195 at day 16 post-MCAO	• No significant effect of tDCS on NSC proliferation measured by [^18^F]FLT PET but significant improvement measured by IHC • **[**^**11**^**C]-R-PK11195 not quantified, but increased uptake in the peri-infarcted area and thalamus (secondary damage)** • Cathodal tDCS significantly increased the M1-polarization of microglia	T2 MR at day 2 post-MCAO to assess the success of MCAO, TSPO-PET confirmed by iba+ IHC	[100]
[^18^F]DPA-714 [^18^F]FSPG (system xc^-^) [103]	Better understanding of system xc^-^ in NI vs TSPO as reference biomarker for NI in stroke	90-min intraluminal MCAO in male SD rats (300 g)	Before and at 1, 3, 7, 14, 21 and 28 days post-MCAO	• [^18^F]FSPG: increased uptake peaking at days 3 to 7, decreasing from days 14 to 28 post-MCAO • **[**^**18**^**F]DPA-714: peak uptake at day 7, decreasing from day 14 post-MCAO** • **xc**^**-**^ **inhibitors reduced [**^**18**^**F]DPA-714 uptake at 7 days post-MCAO**	T2 MR 24 h post-MCAO to assess infarct size, PET data confirmed by IHC; system xc^-^ inhibitors reduced expression of M1 (TSPO, CCL2, TNF and iNOS) and increased arginase (M2) biomarkers	[97]
[^11^C]-R-PK11195 [^18^F]FLT (NSC proliferation)	Investigate the role of TLR4 in neurogenesis and inflammation	Proximal (MCA bifurcation) in 2–3-months-old male C57BL/10 J (TLR4^+/+^) mice and distal MCAO (posterior branch) in C57BL/10ScNJ (TLR4^-/-^) mice	2, 7 and 14 days post-MCAO	• **Modest increase in [**^**11**^**C]-R-PK11195 uptake 2 days post-MCAO in TLR4**^**+/+**^**, not in TLR4**^**-/-**^ **mice** • **Similar increase in [**^**11**^**C]-R-PK11195 uptake in TLR4**^**+/+**^ **and TLR4**^**-/-**^ **mice at 7 days** *In TLR4*^*-/-*^ *mice:* • higher uptake of [^18^F]FLT • **Decreased uptake of [**^**11**^**C]-R-PK11195** *than that in the TLR4*^*+/+*^ *mice*	T2 MR 24 h post-MCAO to assess infarct size, PET data confirmed by IHC	[98]
[^18^F]DPA-714	Investigate the role of NI in SAH	Intraluminal ACA puncture in male Wistar rats (300–350 g)	2 days post-SAH	• **Trend (*p*** **= 0.519) to increase in [**^**18**^**F]DPA-714 SUVr in SAH vs sham rats** • **Strong correlation between [**^**18**^**F]DPA-714 uptake and grade of SAH bleed**	In vivo PET data confirmed by [^3^H]-R-PK11195 autoradiography and IHC	[82]
[^11^C]PBR28	Better understanding of NI in stroke disease mechanisms	90-min left MCAO (M2 segment) by microwire tip insertion through the ventral tail artery in SD rats (362±28 g)	1, 4, 7 and 14 days post-MCAO	• **Significant increase in [**^**11**^**C]PBR28 uptake in the infarct at day 4, 7 and 14 vs day 1 and vs contralateral side, decreasing at day 14** • **No significant change in the contralateral side**	T2 MR before each PET acquisition, in vivo PET findings confirmed by CD11b, CD68, GFAP and TSPO IHC	[83]
Four new [^18^F]-labelled acetamidobenzoxazolone TSPO compounds	Selection of best TSPO tracer candidate	30-min intraluminal MCAO in male SD rats (7 weeks old, 220−240 g)	7 days post-MCAO	• **Best compound: 2-(5-(6-[**^**18**^**F]fluoropyridin-3-yl)-2-oxobenzo[d]oxazol-3(2H)-yl)-N-methyl-N-phenylacetamide (compound d) with and ipsi-/contralateral ratio of 4.20 ± 0.37 and BP**_**ND**_ **of 2.33 ± 0.25**	**C6 glioma model in rats (ipsi-/contralateral ratio, 4.20±1.09; BP**_**ND**_**, 2.29±079)**; ARG confirmed in vivo PET	[74]
[^11^C]-R-PK11195 and [^11^C]NE40 (CB2 receptors) [104]	Evaluation of [^11^C]NE40 and CB2 receptor as marker of early microglial activation	Photothrombotic left MCAO in 8-weeks-old male SD rats (250–300 g)	24h post-MCAO	• **No significant increase in [**^**11**^**C]-R-PK11195 uptake** • Significant increase in [^11^C]NE40 BP_ND_ and in the ipsilateral frontal (1.79±0.99) and parietal (1.27±0.77) cortices	IHC confirmed the absence of TSPO 24 h post-MCAO; CB2 staining colocalized with a variety of cells positive for CD11b, NeuN and NG2 staining	[93]
[^18^F]VUIIS1008 [^18^F]DPA-714	Comparison of new TSPO tracer [^18^F]VUIIS1008 with [^18^F]DPA-714	90-min intraluminal MCAO in male SD rats (296±9 g)	Before and at 1, 3, 7, 14, 21 and 28 days post-MCAO	• **[**^**18**^**F]VUIIS1008 and [**^**18**^**F]DPA-714 displayed similar uptake in the infarct, peri-infarct and contralateral brain regions** • **[**^**18**^**F]VUIIS1008 displaced to contralateral level by unlabelled VUIIS1008 with DPA-714 at 1 mg/kg**	• Infarct size confirmed to be similar for both tracers. • Microglial/macrophage cellular localization of TSPO confirmed by CD11b IHC	[76]
[^18^F]F-DPA	Radiochemistry and preliminary evaluation of a novel TSPO tracer: [^18^F]F-DPA ([^18^F]DPA-714 derivative)	30-min intraluminal MCAO in SD rats	7 days post-MCAO	• **Ipsi- to contralateral ratio: 4.26±0.34 at 90min post-injection** • **Ipsilateral BP**_**ND**_**: 4.02±1.32**		[78]
[^18^F]GE180	Evaluate microglial activation after global perinatal hypoxic injury	Hypoxia resuscitation in newborn Noroc piglets (12–36 h old) + 1 positive control (LPS injected)	Baseline and 0–5 h, 6–8 h, 24–26 h and 29–32 h after hypoxia/ resuscitation	• **Hypoxia resuscitation did not increase [**^**18**^**F]GE180 uptake in any of the brain regions measured** • **LPS induced an increase in [**^**18**^**F]GE180 uptake in the LPS-injected basal ganglia**	Post-mortem T1 and T2 MRI at the end of the experiment for anatomical co-registration and brain ROI delineation	[81]
[^18^F]5 and [^18^F]6	Development and test of new [^18^F]fluorobenzene ring-based TSPO radiotracers	30-min intraluminal MCAO in male SD rats (7 weeks old, 220–240 g)	*Number of days post-MCAO not specified*	• **[**^**18**^**F]5: ipsi-/contralateral ration, 4.49±0.26; BP**_**ND**_**, 3.42±0.23** • **[**^**18**^**F]6: ipsi-/contralateral ration, 2.98±0.37; BP**_**ND**_**, 1.76±0.21** • **Both have a better signal-to-noise ratio than that of [**^**11**^**C]-R-PK11195** • **[**^**18**^**F]5 is the best candidate**		[75]
[^18^F]DPA-714 [^18^F]IAM6067 (S1R tracer) [92]	Comparison of in vivo PET with MALDI-MS imaging on long-term consequences of stroke on ND (S1R] and NI (TSPO)	70-min distal left MCAO with CCAO in male Wistar rats (350 g)	3 months post-MCAO	• **No change in [**^**18**^**F]DPA-714 uptake** • **No change in [**^**18**^**F]IAM6067 uptake** MALDI-MS imaging: • Downregulation of phosphatidylcholine in the ischemic scar • Upregulation of lysophosphatidylcholine and sphingomyelin in the ischemic scar	T2 weighted (T2W) MRI at 48 h post-stroke confirmed the presence of infarct	[91]
[^18^F]DAA1106	Automation of [^18^F]DAA1106 synthesis	30-min intraluminal MCAO in male SD rats (7 weeks old, 220–240 g)	6–8 days post-MCAO	• **AUC**_**20–60min**_ **in the striatum:** **° Ipsilateral: 44.7±7.7** **° Contralateral: 23.7±0.1** • **Ipsi-/contralateral ratio: 1.9±0.3** • **Pretreatment with unlabelled PK11195 (3 mg/kg) blocks [**^**18**^**F]DAA1106 uptake**		[105]
[^18^F]DPA-714	Examine longitudinal changes in TSPO after mild ischaemia with selective neuronal loss but without acute infarction	20-min intraluminal MCAO in male Wistar rats (9–10 weeks old, approx. 300 g)	• 2 and 7 days post-MCAO • ARG at day 1, 2, 3 and 7 post-MCAO	• **VT values post-MCAO:** **° Day 2: ipsilateral, 12.9±1.9; contralateral, 9.7±1.3; ipsi-/contralateral ratio, 1.3±0.2** **° Day 7: ipsilateral, 12.4±2.5; contralateral, 7.8±0.8; ipsi-/contralateral ratio, 1.6 ± 0.2**	• [^18^F]DPA-714 in vitro ARG confirmed a significant increase in TSPO binding from day 1, increasing up to day 7 post-MCAO • Iba1 and GFAP IHC confirmed the presence of activated microglia and astrogliosis • Nissl staining confirmed selective neuronal loss	[84]
[^18^F]DPA-714	Evaluate the effects of BMSC administration on neuroinflammation after transient MCAO	90-min intraluminal MCAO in male F344/NSIc rats (250–270 g)	3 and 10 days post-MCAO	• **BMSC administration does not decrease max. and mean SUV 3 days post-MCAO** • **Significant decrease in max. and mean SUV by BMSC administration at 7 days post-MCAO**	• BMSC administration decreases infarct volume • [^18^F]DPA-714 in vitro ARG confirmed in vivo PET data • BMSC administration decreases the number of CD8α+ T cells and CD68 microglia in the infarct and peri-infarct areas	[101]
[^18^F]VUIIS1018A	Evaluation of new TSPO tracer [^18^F]VUIIS1018A	30-min intraluminal MCAO in male SD rats (7 weeks old, 220–240 g)	7–9 days post-MCAO	• **Ipsilateral [**^**18**^**F]VUIIS1018A uptake at 0.73 SUV vs contralateral uptake at 0.20 SUV at 60 min post-injection** • **Ipsi-/contralateral ratio: 3.5**	[^18^F]VUIIS1018A in vitro ARG and TSPO IHC confirmed in vivo PET data	[77]
[^11^C]DPA-713 [^18^F]GE-180	Head-to-head comparisons of [^11^C]DPA-713 and [^18^F]GE-180	Permanent distal MCAO in 3-months-old female C57BL/6J mice	2, 6 and 28 days post-MCAO	**Ipsi-/contralateral ratio:** • **At 2 days, significant increase for [**^**11**^**C]DPA-713 (1.22) but not for [**^**18**^**F]GE-180 (1.10)** • **Significant increase for [**^**11**^**C]DPA-713 and [**^**18**^**F]GE-180 at 6 days (2.20 and 2.06, respectively) maintained at 28 days (1.67 and 1.64, respectively)**	• T2 MRI for ROI delineation • Ex vivo [^11^C]DPA-713 and [^18^F]GE-180 ARG confirmed the PET data • CD68 and GFAP IHC confirmed the presence of activated microglia and astrogliosis in the infarct	[80]
[^18^F]FEPPA	Test the ability of TSPO to detect MHCII^+^ microglia in WM post-stroke	Intrastriatal injection of ET1 in male Fischer 344 strain (11–14 months)	Baseline and 7 and 28 days post-MCAO	**TSPO infarct/cerebellum ratio:** • **Baseline: 0.94±0.16** • **Day 7: 2.10±0.78** • **Day 28: 1.77±0.35** • **Similar change in peri-infarct WM but not contralateral WM**	• T2 MRI for ROI delineation and co-registration • IHC for TSPO confirmed the presence of microglial activation in infarct and peri-infarct WM at day 7 • No increase in TSPO in peri-infarct WM at day 28 • Contralateral NI detected by OX6 MHC IHC at day 28 was not detected by PET imaging of TSPO/iNOS IHC	[86]
[^18^F]DPA-714 [^18^F]BR-351 (MMPs) [106] [^99m^Tc]HMPAO (CBF)	Investigate NI through TSPO and MMP PET imaging in stroke	30-min intraluminal MCAO in male C57BL/6 mice (3–4 months old, 22–25 g)	24 to 48 h and 7±1, 14±1 and 21±1 days post-MCAO	• **No increase in TSPO at 24–48 h** • **Significant increase at 7, 14 and 21 days, peaking at 14 days** • **Localization of increased [**^**18**^**F]DPA-714 uptake is larger than T2w-MRI infarct, not the case for [**^**18**^**F]BR-351** • **Volumetric analysis: MMP expression peaks at 24–48 h and has a different spatio-temporal distribution than TSPO** • MMPs increased at all time points, with an increase in the contralateral side at day 21	• Severity of stroke confirmed by [^99m^Tc]HMPAO and T2 MRI • TSPO and MMP9 IHC confirmed PET results	[95, 94]
[^18^F]CPFPX (A1AR) [107] [^18^F]DPA-714 [^18^F]FLT	Better understanding of the role of A1ARs on ischaemic damage	90-min intraluminal MCAO in male SD rats (8 weeks old, 304±7.1 g	•[^18^F]CPFPX PET at baseline and 1, 3, 7, 14, 21 and 28 days post-MCAO • [^18^F]DPA-714 PET at 7 days post-MCAO	• Decrease in [^18^F]CPFPX uptake at day 1 returning to baseline level at day 3, gradually decreasing to reach significant differences vs baseline at days 21 and 28 post-MCAO • **Treatment with A1AR agonist ENBA reduced [**^**18**^**F]DPA-714 uptake** and [^18^F]FLT uptake infarct size and improved the neurological score	• IHC showed an increase in A1ARs at days 3 and 7 returning to baseline level 14 days post-MCAO localized on microglia and macrophages • Treatment with A1AR agonist ENBA reduced CD11b/TSPO and Ki67 IHC staining in the infarct	[99]

*TSPO-PET tracers and associated results are in bold.

Abbreviations: *[*^*18*^*F]5*, N-(4-[18F]fluorobenzyl)-N-methyl-2-(7-methyl-8-oxo-2-phenyl-7,8-dihydro-9H-purin-9-yl)acetamide; *[*^*18*^*F]6*, 2-(5-(4-[^18^F]fluorophenyl)-2-oxobenzo[d]oxazol-3(2H)-yl)-N-methyl-N-phenylacetamide; *[*^*18*^*F]FDG*, fluorodeoxyglucose; *[*^*18*^*F]FLT*, fluorothymidine; *[*^*18*^*F]FSPG*, (4S)-4-(3-18F-fluoropropyl)-L-glutamate; *[*^*99m*^*Tc]HMPAO*, ^99m^Tc-D,L-hexamethylene-propyleneamine oxime; *A1AR*, adenosine A1 receptors; *ACA*, anterior cerebral artery; *ARG*, autoradiography; *BMSC*, bone marrow stromal cell; *CCAO*, common carotid artery occlusion; *CB2*, cannabinoid type 2 receptors; *CBF*, cerebral blood flow; *ET1*, endothelin-1; *IHC*, immunohistochemistry; *MALDI-MS*, matrix-assisted laser desorption/ionization mass spectrometry; *MCAO*, middle cerebral artery occlusion; *MHCII*, major histocompatibility complex class II; *MMPs*, matrix metalloproteinases; *nAChR*, nicotinic acetylcholine receptor; *ND*, neurodegeneration; *NI*, neuroinflammation; *NSC*, neural stem cells; *S1R*, sigma-1 receptor; *SAH*, subarachnoid haemorrhage; *SD*, Sprague Dawley; *SUVr*, standard uptake value ratio; *tDCS*, transcranial direct current stimulation; *TLR4*, toll-like receptor; *WM*, white-matter; *xc*^*-*^, cystine-glutamate antiporter system

To test new TSPO tracers, stroke models present the advantages of being clinically relevant and to induce a strong TSPO^+^ microglial activation that has been demonstrated both preclinically [8, 11] and clinically [57, 58, 60]. In this context, Boutin et al. used a transient model of middle cerebral artery occlusion (MCAO) in rats to evaluate the then-new TSPO tracer [^18^F]GE-180 [67] and demonstrated a superior uptake, ipsi-/contralateral ratio and BP_ND_ for [^18^F]GE-180 when compared to [^11^C]-R-PK11195. This preclinical study and another using LPS-induced milder neuroinflammation [40] positively demonstrated the benefit in terms of the signal-to-noise ratio of [^18^F]GE-180 vs [^11^C]-R-PK11195. However, clinical studies demonstrated that, for reasons that remain unclear, the brain pharmacokinetics of [^18^F]GE-180 were slower – i.e. more than one could anticipate – and less favourable in man as those observed in rat. Despite this observation, several studies have recently shown the value of this tracer to measure neuroinflammation in vivo [68–73]. In the same line, Fujinaga et al. used a stroke model in rats to test and select new TSPO tracer candidates and identified that amongst those 2-(5-(6-[^18^F]fluoropyridin-3-yl)-2-oxobenzo[d]oxazol-3(2H)-yl)-N-methyl-N-phenylacetamide (compound d in [74]) and [^18^F]5 (N-(4-[18F]fluorobenzyl)-N-methyl-2-(7-methyl-8-oxo-2-phenyl-7,8-dihydro-9H-purin-9-yl)acetamide) [75] were the best candidates with better signal-to-noise ratio than [^11^C]-R-PK11195. Other TSPO tracers have been evaluated using stroke models, such as [^18^F]VUIIS1008 [76], [^18^F]VUIIS1008A [77] and [^18^F]F-DPA [78]. Generally, those new tracers showed potential with a better signal-to-noise ratio than that of [^11^C]-R-PK11195 that they aim to replace. However, the direct comparison of [^18^F]VUIIS1008 with [^18^F]DPA-714 revealed that [^18^F]VUIIS1008 yielded no gain in term of signal-to-noise ratio when compared to [^18^F]DPA-714, with similar ipsi- to contralateral ratio and BP_ND_ values. When comparing, a posteriori*,* data reported for [^18^F]VUIIS1008A, [^18^F]VUIIS1008 and [^18^F]DPA-714, the same group seemed to indicate that it was also the case for [^18^F]VUIIS1008A [77]. However, in a more recent study using [^18^F]VUIIS1008 and [^18^F]DPA-714, a significant increase in uptake from day 3 post-MCAO was reported, peaking at day 7 and remaining elevated up to day 14 when compared to baseline [76], in line with previous reports [65]. The same observation can be made for [^18^F]F-DPA in terms of binding characteristics and uptake levels. However, its lower metabolism rate when compared to [^18^F]DPA-714 is an interesting feature of this new tracer [79]. Also performing a direct comparison between 2nd generation TSPO tracers, Chaney et al. [80] compared [^11^C]DPA-713 and [^18^F]GE-180 in a model of permanent MCAO in mice. In this study, they showed that, 1 day post-MCAO, [^11^C]DPA-713 uptake was significantly increased (+22%) whereas [^18^F]GE-180 uptake was not (+10%); 6 days post-MCAO, both tracers exhibited similar uptake increases (+120% and +106%, respectively) which remained elevated up to 28 days post-MCAO (+67% and +64%, respectively) [80].

The other major use of TSPO imaging in stroke models is to better understand the role of various neuroinflammation processes following experimental stroke. In that perspective, some studies used TSPO-PET imaging to better characterize the temporal and anatomical evolution of neuroinflammation in various models of experimental stroke, some of them new. De Lange et al. [81] used [^18^F]GE-180 to evaluate acute neuroinflammation in a model of hypoxia resuscitation in newborn piglets. Considering the numerous reports showing that it takes approximately 3 days for microglia to proliferate and TSPO expression to increase in stroke and LPS model of neuroinflammation, unsurprisingly this study did not detect any significant change in [^18^F]GE-180 between 5 and 32 h post-hypoxia. Similarly, in a model of subarachnoid haemorrhage (SAH) in rats, Thomas et al. [82] showed only a trend (*p* = 0.0519) in an increase in [^18^F]DPA-714 uptake 2 days post-SAH. In a very clinically relevant model of focal temporary MCAO using a minimally invasive technique through the insertion of a microwire tip via the ventral tail artery, Toth et al. [83] demonstrated significant increases in [^11^C]PBR28 uptake in the infarct at day 4, 7 and 14 post-MCAO, although [^11^C]PBR28 uptake started to decrease at day 14. Their results are in line with other studies using more conventional models of stroke in rats [76, 65] such as those developed by Miyajima et al. [84] described a significant increase in [^18^F]DPA-714 uptake in the infarct from day 2 maintained up to day 7 post-MCAO (20-min intraluminal MCAO). Taken altogether, these results confirmed that in these models of acute brain injury (permanent or temporary MCAO, SAH or hypoxia), the microglial/macrophage infiltration responsible for the TSPO increase takes approximately 3 days to become detectable, peaks about 1 week after injury and then slowly decreases in intensity afterwards.

On top of characterizing the temporal evolution of TSPO expression after stroke, some studies used TSPO-PET tracers combined with other PET tracers or other imaging methods to investigate further processes in stroke. Walter et al. combined [^11^C]-R-PK11195 PET with USPIO MR imaging to differentiate nonphagocytic (TSPO^+^ only) vs phagocytic (TSPO^+^ & USPIO^+^) neuroinflammatory cells in a permanent microsphere-induced model of MCAO in rats 6–7, 26–27 and 55–56 days post-stroke [85]. Such combination of techniques allowed the authors to determine that brain regions with early phagocytic signal (USPIO^+^) at day 7 will irremediably evolve into necrotic tissue, whereas tissue exclusively positive for TSPO will remodel but remains viable thereafter (days 27 and 55 post-stroke). Looking at white matter damage after stroke, Al-Khishman et al. used [^18^F]FEPPA TSPO-PET imaging and reported a significant increase at 7 and 28 days in the striatal infarct and peri-infarct white matter (WM) [86]. In the contralateral WM, they were able to detect MHCII^+^ cells by either [^18^F]FEPPA TSPO-PET or TSPO IHC. In a way, this highlights the fact that TSPO is an imperfect surrogate marker of activated microglia and may be unable to detect certain phenotype of microglia.

Combining [^11^C]-R-PK11195 and [^18^F]FDG, Backes et al. [87] performed a study investigating the potential contribution of neuroinflammation to [^18^F]FDG signal in the same model as Walter et al. [85]. Not unexpectedly, they found that microglial activation and macrophage infiltration could indeed be the source of a significant [^18^F]FDG uptake at day 7 post-stroke in the peri-infarct region where [^18^F]FDG uptake was initially significantly decreased at day 1. These observations are in line with previous use of [^18^F]FDG to image inflammation [88–90], although very unspecifically especially in the brain where the basal [^18^F]FDG uptake is very high mostly due to neuronal activity. They concluded that in such brain regions, where metabolic supply was already decreased and likely insufficient, the contribution of neuroinflammation to the [^18^F]FDG signal might actually mask even more severe hypometabolism. Also looking at complementing PET imaging with another tracer and method, Henderson et al. [91] combined PET imaging with [^18^F]DPA-714 and the newly developed sigma-1 receptor (tracer [^18^F]IAM6067 [92] and post-mortem mass spectrometry imaging (MALDI-TOF) to look at the long-term consequences of distal MCAO. These authors did not find any changes in [^18^F]DPA-714 and [^18^F]IAM6067, suggesting that 3 months post-MCAO, neuroinflammation had resolved and that neuronal loss directly in or at a distance of the infarct was undetectable using S1R PET imaging. MALDI-TOF MS however revealed distinctive changes in the peri-infarct scar with a decrease in phosphatidylcholines, which are otherwise found in the healthy tissue, whereas sphingomyelin and lysophosphatidylcholine were significantly decreased in the same region. The MALDI-TOF MS combined with the TSPO-PET data suggest that neuroinflammation had fully recessed in the peri-infarct region 3 months after MCAO where scarification and repair were taking place. Using [^11^C]-R-PK11195 PET imaging as a reference for neuroinflammation imaging, Hosoya [93] tested the potential of the cannabinoid type 2 receptor (CB2) as an early marker for neuroinflammation 24 h post-MCAO. They reported a significant increase in [^11^C]NE-40 uptake but no change in [^11^C]-R-PK11195 uptake. Further immunohistochemistry experiments revealed that most of the CB2^+^ cells were microglia (CD11B^+^) together with some neuron-glial antigen 2-positive (NG2^+^) cells putatively identified as monocytes, supporting CB2 and [^11^C]NE-40 as a potential candidate of early microglial activation, although in that case, the exact phenotype of the microglia remains to be established. Finally, investigating the relationship between microglial activation and metalloproteases (MMP), Zinnhardt et al. [94] and Barca et al. [95] investigated TSPO expression with [^18^F]DPA-714 and MMP levels with [^18^F]BR-351. These studies showed a differential temporal expression between TSPO and MMP; MMP levels increased early (days 1–7 post-MCAO) and remained elevated while TSPO levels increased later (days 7–21 post-MCAO) in the infarct. Interestingly, MMP were also significantly increased in the contralateral side at 21 days, where no change in TSPO could be detected. By refining the differential analysis of the two tracers and comparing it with the T_2_^_^weighted MR map of the infarct, Barca et al. [95] concluded that this differential pattern likely represented a different phase of acute inflammation in the infarct followed by remodelling in the peri-infarct and contralateral side.

Focusing more on therapeutic approaches in relation with neuroinflammation, Martin et al. published various papers using TSPO as reference for neuroinflammation imaging in which they examine the involvement of α4β2 nicotinic acetylcholine receptor (nAChR) [96], the cystine-glutamate antiporter system xc^-^ [97], toll-like receptor 4 (TLR4) [98] and the adenosine A1 receptors (A1AR) [99] in the regulation of neuroinflammation after stroke. Overall, they described a very consistent time course of TSPO expression across studies and models. They also demonstrated that 2[^18^F]-fluoro-A85380 (α4β2 nAChR) and [^18^F]FSPG (system xc^-^) uptakes were maximum at 7 and 3–7 days post-stroke, respectively, and decreased thereafter, paralleling TSPO levels. Conversely, [^18^F]CPFPX (A1 adenosine receptor, A1AR) followed an inverse pattern to TSPO levels along time with an early decrease 24 h post-stroke returning to baseline at day 3 followed by a subsequent progressive decrease reaching significance vs baseline at days 21–28 post-MCAO. It must be noted that there was a slight discrepancy between the temporal expression of the A1AR measured by PET and immunohistochemistry as for the latter, there was no decrease observed at day 1 and no significant decrease at days 21–28 vs baseline. Altogether, these studies demonstrated that these systems are altered following stroke. More importantly, these studies also demonstrated that modulation of the α4β2 nAChR by the selective antagonist DhβE increased [^11^C]-R-PK11195 uptake [96], whereas treatment with inhibitors of the system xc^-^ [97] or the A1AR agonist ENBA [99] significantly decreased neuroinflammation as measured by [^18^F]DPA-714 uptake and immunohistochemistry. In the same line, [^11^C]-R-PK11195 PET imaging showed that neuroinflammation was reduced in TLR4^-/-^ mice 2 days but not 7 days post-MCAO [98]. Similarly, Braun et al. [100] investigated the effect of transcranial direct current stimulation (tDCS) on neuroinflammation and neural stem cell (NSC) proliferation 16 days post-MCAO and showed no effect of tDCS on NSC proliferation as measured by [^18^F]FLT PET but did observe an effect when measured by immunohistochemistry; [^11^C]-R-PK11195 was unfortunately not quantified, and only a noticeable uptake in the peri-infarct and thalamus was reported. Finally, Tan et al. [101] evaluated the therapeutic effect of intravenous administration of bone marrow stromal cell (BMSC) to modulate the inflammatory response to stroke. They showed that [^18^F]DPA-714 uptake increased 3 days post-MCAO similarly in vehicle and BMSC-treated rats, but 7 days post-MCAO, [^18^F]DPA-714 uptake was significantly reduced in BMSC-treated animals compared to vehicle animals. The in vivo PET results and therapeutic effect of the BMSC treatment were confirmed by autoradiography and were correlated with a decrease in infarct volume as well as a decrease in the number of CD8α+ T cells and CD68 microglia in the infarct and peri-infarct areas.

## Neuroinflammation in models of chronic neurodegenerative diseases

### Alzheimer’s disease (AD)

Similarly to stroke and other neurodegenerative diseases, neuroinflammation emerged as a potential essential player in the pathophysiology of AD in the 1990s [108, 109], although its presence was already acknowledged by Alois Alzheimer himself when he first described the neuropathological features of AD [110–112].

In 2019, Chaney et al. published a review of neuroinflammation imaging in Alzheimer diseases [113] that included preclinical references up to the work of Lopez-Picon et al. [114]. For this paragraph, we used the following PubMed search, “(omega* OR TSPO OR PBR OR PK11195) AND (PET OR positron) AND (brain OR cereb*) AND (Alzheimer) AND (rat OR mouse OR mice OR animal* OR experimental OR pig OR gerbil OR rabbit OR guinea OR model OR dog OR cat) AND (English[ lang])”, including all references since 2018; this search returned 37 records including 6 reviews. After exclusion of the reviews, clinical papers and non-PET imaging manuscripts returned by this search, 8 original research papers that included in vivo TSPO imaging were kept in this review and are summarized in Table 4.

**Table 4:** Overview of the preclinical TSPO-PET/SPECT imaging studies in Alzheimer’s disease models (published since 2019 [113])

PET tracer *	Rational	AD model	Imaging time points	Main imaging findings	Additional readouts	Ref.
[^18^F]GE180 [^15^O]H_2_O (CBF)	Investigate the short-term effects of space radiation (model by ^56^Fe irradiation at 4 months of age) on brain ageing and AD	3.5-months-old male and female APPswe/PS1dE9 Tg mice	At baseline (3.5 month old) and 1.5–2 month post-irradiation	• **Irradiation induced a small decrease in the initial (0–20 min post-inj.) and late [**^**18**^**F]GE180 uptake (20–60 min post-inj.) in Tg female mice only** • **No changes in any of the other groups** • No change in CBF	• Irradiation induces a reduction in Aβ and CD68 IHC staining in Tg female only • No effect on TSPO, Iba-1 or GFAP staining in any of the group	[125]
[^18^F]GE-180 [^18^F]florbetaben (Aβ) [126, 127]	Establish serial small-animal PET as a tool for therapy monitoring of the new model of AD App^NL-G-F^ mice	Males and females App^NL-G-F^ knock-in [128] and WT mice	2.5, 5, 7.5 and 10 months of age	• **Increased [**^**18**^**F]GE-180 SUVr in the cortex from 5 m and the hippocampus from 7.5 m in Tg mice, increasing further at 10m** • Similar spatiotemporal pattern for an increase in [^18^F]florbetaben in Tg mice • **Correlations between tracer uptake ([**^**18**^**F]GE-180 SUVr and [**^**18**^**F]GE-180/[**^**18**^**F]florbetaben ratio) and spatial learning performance in the right entorhinal/piriform cortex and the right amygdala (NS in left ROIs)**	IHC for fibrillar and non-fibrillar Aβ and neuroinflammation markers (Iba1, TREM2) confirmed PET results	[116, 117]
[^125^I]-CLINDE	Investigate the relationship between TSPO and Aβ load in AD mice	Female triple transgenic (3×TgAD, APP_swe_, PS1_M146V_ and Tau_P301L_) and C57B1/6J-Sv129 control mice	21 months of age	• **Increase [**^**125**^**I]-CLINDE uptake in TG vs WT mice** • **Significant spill over in the hippocampus from the nearby ventricular zones** • **[**^**125**^**I]-CLINDE uptake in the pituitary, lateral ventricles and cerebellum is specific (29.4±7.6 to 61±3.9% displacement by the unlabelled CLINDE)**	**[**^**125**^**I]-CLINDE** and [^125^I]DRM106 (Aβ) [129] ARG at 1, 6, 12 or 21 months confirmed in vivo SPECT result with **[**^**125**^**I]-CLINDE** and showed that **TSPO increase precedes Aβ load in most of the hippocampal substructures**	[124]
[^11^C]-R-PK11195 [^11^C]NE40 (CB2) [104]	Investigate microglial status in brain senescence	Male SAMP10 mice	5 and 15 weeks of age	• **No change in [**^**11**^**C]-R-PK11195 at any ages** • No change in [^11^C]NE40 SUVr at 5 weeks • [^11^C]NE40 SUVr > [^11^C]-R-PK11195 SUVr at 15 weeks	• Increase in CB2/Iba1^+^ cells at 15 weeks suggesting an anti-inflammatory phenotype • TSPO IHC consistent with low/basal level TSPO expression measured by PET	[123]
[^18^F]FEPPA	Test the ability of TSPO to detect MHCII^+^ microglia in WM in a rat model of AD	F344 and TgAPP21 rats	11–14 months of age	• **No significant increase in [**^**18**^**F]FEPPA SUVr in previously reported** **[**121**]** **WM MHCII**^**+**^ **microglia in TgAPP21 rats**		[122, 86]
[^18^F]DPA-714	In vivo assessment of neuroinflammation in AD mice	B6.Cg-Tg (APP_swe_, PSEN1_dE9_) 85Dbo/Mmjax and WT mice	6-7, 9–10, 12–13, and 15–16 months of age	• **No TG vs WT differences at 6–7 and 9–10 months** • **Increase in cortical (+40%) and hippocampal (+60%) [**^**18**^**F]DPA-714 SUVr at 12-13 and 15–16 months**	IHC for Iba1 and TSPO confirmed and correlated with PET data	[120]
[^18^F]F-DPA	Establish the impact of low molar activity tracer on specific brain uptake	Males and females TG APP/PS1-21 and WT mice	9 months of age	• **Significant increase in tracer SUVr in TG vs WT in more brain ROIs with high molar activity [**^**18**^**F]F-DPA** • **High molar activity tracer produced greater (1.5x) differences between TG and WT**	• ARG confirmed the TG vs WT differences in all brain ROIs • Significant differences between low and high molar activity uptake in TG	[115]

*TSPO-PET tracers and associated results are in bold.

Abbreviations: *ARG*, autoradiography; *CBF*, cerebral blood flow; *CB2*, cannabinoid type 2 receptor; IHC, immunohistochemistry; *ND*, neurodegeneration; *NS*, non-significant; *ROI*, region of interest; *SAMP10*, senescence-accelerated mouse prone 10; *SUVr*, standard uptake value ratio; system; *TG*, transgenic; *WT*, wild type

AD animal models need to be aged and are consequently expensive to breed and maintain. In such models and despite its cost, in vivo imaging presents the advantages of reducing the number of animals while increasing statistical power through a longitudinal (i.e. repeated) analysis of the same cohort of animals.

In a rather unusual study, Liu et al. used [^18^F]GE-180 PET to investigate the effect of space radiation on normal brain ageing and on AD between 3.5 and 5 months of age in mice. They found that irradiation had only a very limited effect on brain inflammation, with only a minute reduction in [^18^F]GE-180 uptake in female TG vs nonirradiated mice, no such effect was observed in male mice.

Keller et al. [115] reported the only paper that used the APP/PS1-21 model at 9 months of age to test a new TSPO ligand. In this paper, they wanted to evaluate the impact of low vs high molar activity of [^18^F]F-DPA on brain uptake. This is a relevant technical point to all preclinical PET studies. The molar activity of a tracer should not be neglected when performing PET imaging in small animals such as mice which have a small body weight and blood volume because the presence of a too high concentration of cold ligand (i.e. low molar activity) is more likely to impact the binding and may lead to a poor sensitivity of the measures to pathological changes. In this study, they reported a significant 1.5-fold difference in brain uptake in favour of the high molecular activity. However, it must be noted that in this article, the difference between the low and the high molar activity was particularly large (about 100-fold; 2.25±0.96 GBq/mmol vs 260±110 GBq/mmol), which is an extreme case scenario.

Most of the other studies reviewed here were trying to characterize and understand the temporal and anatomical pattern of neuroinflammation in various models of AD. Sacher et al. [116] and Biechele et al. [117] monitored neuroinflammation in the new App^NL-G-F^ model of AD in mice together with Aβ with [^18^F]florbetaben. In this model, [^18^F]GE-180 TSPO and Aβ levels started to increase at 5 months of age and continue to further increase up to 10 months of age, earlier than in other models such as the APP_swe_×PS1_ΔE9_ [118, 119], suggesting a rather aggressive AD-like phenotype. In agreement with these previous reports [118, 119], Hu et al. [120] reported no changes in [^18^F]DPA-714 uptake in APP_swe_×PS1_ΔE9_ mice between 6 and 10 months of age with an increase in [^18^F]DPA-714 SUVr becoming significant at 12 and 16 months of age.

Now trying to understand the meaning of TSPO signal or lack of it, Al-Khishman et al. [86] tried to determine the sensitivity of PET imaging to detect MHCII+ microglial cells as they did in a model of stroke (see above). Using the TgAPP21 transgenic rat model of AD and [^18^F]FEPPA PET, they showed no significant increase in [^18^F]FEPPA PET uptake in the TgAPP21 rats vs WT at 11–14 months of age, despite the same group demonstrating the presence of MHCII+ cells in previous reports [121, 122]. This observation confirms the one made in the stroke model [86] that TSPO-PET is not able or sensitive enough to detect all phenotypes of activated microglia.

Although related to AD, Yamagishi et al. [123] tried to address the slightly different question of the microglial status during brain senescence using a dual tracer study with [^11^C]-R-PK11195 for TSPO imaging and [^11^C]NE-40 for CB2 imaging in 5- and 15-weeks-old SAMP mice. In this study, the authors showed no TSPO expression at all ages but an increase in CB2 binding and expression at 15 weeks of age compatible with an anti-inflammatory phenotype of the microglia.

Finally, and although the in vivo imaging performed by Tournier et al. [124] is restricted to only 1 time point (21 months old) in the 3×Tg mouse model of AD with [^125^I]CLINDE, the authors interestingly showed a significant spill over signal in the hippocampus due to significant tracer uptake in the ventricles/choroid plexus. Interestingly, this observation highlights the difficulty to perform accurate quantitative in vivo imaging studies in mouse models of AD due to the small size of the mouse brain, also highlighting the importance of selecting fairly large ROIs (such as the whole cortex or whole hippocampus) to limit the impact of partial volume effects in mice brain imaging quantification. This is further illustrated by the fact that the dual autoradiography [^125^I]CLINDE/[^125^I]DRM106 (Aβ) allowed Tournier et al. [124] to demonstrate that increase in TSPO binding (from 6 months in the subiculum) preceded Aβ increase and that these increases affected various part of the hippocampus differentially (subiculum then antero-dorsal and dorsal hippocampus and finally ventral hippocampus).

### Parkinson’s disease

In 1988, McGeer and colleagues reported for the first time the presence of reactive microglia in the brains of Parkinson’s disease (PD) patients [130]. They suggested what is now generally achieved that the immune system plays an active role and that neuroinflammation is not just a consequence of the ongoing neurodegeneration [131, 132]. Nevertheless, the interplay between cytokines, neurodegeneration, and protein aggregation as cause or consequence remains largely unknown today [133, 134, 131]. Over the last two decades, neuroinflammation in PD has been a strongly growing research area, but only few TSPO-PET studies [135–138] or TSPO-autoradiography [139–141] studies have been reported in preclinical models. In addition, these studies generally report on the feasibility of monitoring brain inflammation but less on the mechanistic of neuroinflammation in PD. Recently, Belloli and colleagues [142] have provided a detailed overview of translational imaging studies in PD, including TSPO-PET imaging; complementary to this review, we only focus on TSPO-PET and autoradiography studies in preclinical models of PD. We used the following search in PubMed, “(omega* OR TSPO OR PBR OR PK11195) AND (PET OR positron) AND (brain OR cereb*) AND (Parkinson OR Parkinson's disease OR synuclein) AND (rat OR mouse OR mice OR animal* OR experimental OR pig OR gerbil OR rabbit OR guinea OR primate OR dog OR cat) AND (English[ lang])”, which returned initially 16 records, of which only 6 references referred to TSPO-PET imaging studies in preclinical models. An overview of TSPO-PET in preclinical PD models is shown in Table 5.

**Table 5:** Overview of the preclinical TSPO-PET/SPECT imaging and autoradiography studies in Parkinson’s disease models

PET tracer	Rational	Parkinson model			Imaging time points	Major outcomes	Ref.
		Toxin	Dose	Species			
[^11^C]DAA1106 [^11^C]-R-PK11195	Associate the neuroinflammatory response to 6-OHDA-induced dopaminergic cell loss	6-OHDA	20–24 μg/striatum	Rat	21 days post-injection	Progressive TH^+^ cell loss in STR and SN paralleled with microglial activation; this coincided with positive TSPO-PET signal in SN and STR 3 weeks post-striatal lesion	[144, 39]
[^18^F]DPA-714	Compare a new microglial-specific ligand targeting P2X7 with a well validated TSPO-ligand in an acute (and chronic) PD model	6-OHDA	24 μg/striatum	Rat	4–28 days post-injection	In the 6-OHDA model, maximal TSPO signal was observed in the STR 7 days, and in SN 14 days post-lesion. Uptake decreased until it disappeared in STR 28 day post-injection but not in SN	[139]
[^125^I]CLINDE, [^3^H]-R-PK11195	Longitudinal monitoring of 6-OHDA-induced neuroinflammation and neurodegeneration	6-OHDA	10 μg/striatum	Rat	3–56 days post-injection	Maximal TSPO signal in the STR 7 days and in SN 14 days post-lesion, then declining until it disappears 8 weeks post-injection	[140]
[^18^F]DPA-714	Evaluate with bioluminescence the migration of progenitor cells in a response to neurodegeneration-induced neuroinflammation, monitored with in vivo PET imaging	6-OHDA	2 μl of 5 mg/kg	Mice	7, 14, 21 days post-injection	6-OHDA-induced neuroinflammation-associated neurodegeneration, both detectable by PET imaging. However, this did not result in any migration from the subventricular zone	[145]
[^11^C]-R-PK11195	Characterize the distribution of TSPO bindings sites in the brain and evaluate the feasibility of [^11^C]-R-PK11195 to monitor this binding in healthy, MPTP-treated, and MPTP+grafted brain	MPTP	*Not specified*	Landrace pigs and Göttingen minipigs	Baseline and 2 weeks after MPTP and 3 months after grafting	Despite a high expression of TSPO-binding sites in the porcine brain, [^11^C]-R-PK11195 has a too low specificity in vivo	[147]
[^11^C]-R-PK11195 [^18^F]FEPPA	Monitor early dopaminergic changes and early glial response in a low-dose MPTP model	MPTP	Chronic intoxication 0.1/0.2, 0.6/0.8 mg/kg (IV, IM)	NHP	At baseline and during intoxication	TSPO-PET coincides with a decrease in striatal VMAT2; earlier and robust TSPO-PET signals result in earlier and more severe parkinsonism	[138, 148]
[^11^C]-R-PK11195	Evaluate the role of TREM2 in the regulation of microglia to acute neurotoxic response	MPTP	4x 20 mg/kg (IP)	Mouse *(TREM2*^*-/-*^ *vs WT)*	1–7 days post-injection	TSPO-PET signal gradually increased in STR and SN, reaching significance in STR 2 days after intoxication; TREM2^-/-^ mice showed an earlier increment of [^11^C]-R-PK11195 binding and a significant increase of IL-4	[149]
[^11^C]PBR28	Evaluate the impact of an acute peripheral inflammatory response on the progression of Parkinson’s disease	LPS	Systemic	Rat *(LRRK2 p.G2019S vs WT)*	10 months post-injection	Systemic LPS treatment caused inflammation in the brain, detectable with TSPO-PET. LPS-treated LRRK2 animals exhibited significantly increased neuroinflammation in the cortex and ventral regions compared to control animals	[22]
[^18^F]FEPPA	Evaluate the capability of [^18^F]FEPPA to detect a neuroinflammatory response in a PD model of primary neurotoxic microglial activation	6OHDA LPS	30 μg/striatum (unilateral) Systemic	Rat	2 days post-injection 4 h post-injection	Systemic inflammation induced after 6OHDA-induced neuroinflammation results in higher expression of the pro-inflammatory cytokines IL-1β and TNF-α ipsilateral to the lesion, which correlates with higher TSPO-PET signal in the lesion, as detected with [^18^F]FEPPA	[146]
[^11^C]-R-PK11195	Validation of prostaglandin 2 (PGJ2) infusion in the SN as a PD model of chronic inflammation	PGJ2	16.7 μg/SN	Mouse	7 days post-injection	Chronic PGJ2 administration induces slow-onset PD-like pathology with localized neuroinflammatory response localized in the SN	[155]
[^18^F]DPA-714	Characterization of neuroinflammatory response in a rat model of progressive dopaminergic degeneration	AAV-hα-syn	8 × 10^12^gcp/mL/SNpc (bilateral)	Rat	1, 3 days post-injection; 1–3, 16 WPI	Increased TSPO-PET binding coincided with an increased number of Iba-1^+^ cells in the SN, but astrocytic activation occurred only at a much later stage; significant positive correlation between *BP*_ND_ and the number of Iba-1^+^ cells, but not with GFAP^+^ cells, inverse correlation between *BP*_ND_ and the number of TH^+^ cells; microglial reactivity preceded the reduction of TH^+^ cells	[136]
[^18^F]DPA-714	Compare a new microglial-specific ligand, targeting P2X7, with a well validated TSPO-ligand (in an acute and) chronic PD model	AAV-[A53T]-α-syn	9 × 10^11^gcp/mL/SNpc (unilateral)	Rat	28, 42 days post-injection	Increased TSPO binding in SN between 28 and 42 days and 63 days in the STR; the same model imaged with the microglial PET ligand did not reveal any significant microglial activation; histological data to show the origin of TSPO-PET data are not available	[152, 139]
[^11^C]-R-PK11195	Multitracer PET imaging study to characterize proteasome inhibitor induced model	Lactacystin ICV	200–400 μg	Minipig	Up to 6 months post-administration	Lactacystin leads to the presence of mild neuroinflammation detectable with [^11^C]-R-PK11195; in addition, PET imaging revealed early deficits in the dopaminergic, serotonergic and noradrenergic systems, consistent with Braak staging	[151]
[^11^C]-R-PK11195	Characterization of microglial and dopaminergic response to overexpression of α-syn in the minipig brain	AAV-[A53T]-α-syn	1.04x 10^14^ to 1.16x10^14^ gcp/mL/SNpc (unilateral)	Minipig	4 months post-injection	V_T_ was significantly increased in basal ganglia and cortical regions, in the absence of any motor symptoms or dopaminergic neuronal loss as revealed with in vivo PET or post-mortem studies	[153]

One of the most gold standard models of PD consists of the intracerebral injection of 6-hydoxydopamine (6-OHDA) *– in the substantia nigra (SN), medial forebrain bundle (mfb), or striatum (STR) –* which leads to dose-dependent progressive neurodegeneration and transient neuroinflammation [140, 141, 143, 144]. Cichetti et al. described the time course of tyrosine hydroxylase (TH^+^) cell loss in parallel to microglial activation up to 30 days post-striatal lesion (24μg) and demonstrated dramatic increase in TSPO signal in ventral mesencephalon and striatum 21 days after 6-OHDA lesioning using [^11^C]-R-PK11195 [144]. Maia et al. further developed this work by measuring the time course of the neuroinflammatory response in STR and SN using in vitro binding of [^3^H]-R-PK11195 and ex vivo uptake of [^123^]CLINDE, in parallel to changes in dopamine transporter (DAT) binding and TH^+^ protein levels after 6-OHDA-induced striatal lesion (10 μg) [140]. The TSPO binding in STR peaked 7 days post 6-OHDA induction and then declined progressively at 4 weeks, until disappearing at 8 weeks post-injection. In the SN, a delayed but similar response was observed with maximal TSPO binding at 14 days post-injection. The neuroinflammatory response as measured with TSPO ligands occurred concomitantly with a progressive loss in TH^+^ cells and DAT; however, in this study, it was not identified which glial cell type was TSPO positive. Noteworthy, the same group [141] recently draw a complete metabolomics, neurodegenerative and neuroinflammatory picture of a new variation of the 6-OHDA model (triple striatal injection, 12 μg) through the combination of multiple techniques, amongst which autoradiography using [^3^H]FPA714. In the search for new radioligands more specific for the microglial response, Crabbé and colleagues used the acute transient neuroinflammation of the 6-OHDA model (striatal induction, 24 μg) to compare a new microglial-specific PET ligand targeting the P2YX7 receptor, [^11^C]JNJ717, with the well-validated TSPO ligand [^18^F]DPA-714 [139]. Their TSPO autoradiography study revealed a comparable striatal and nigral TSPO binding pattern as initially described by Maia and colleagues [140]. Surprisingly, the binding of the microglial-specific ligand showed a different temporal pattern than the one observed with the TSPO ligand [^18^F]DPA714 [139]. In the striatum, the microglial response was detectable at 7 days, but maximal around 14 days post-injection, in contrast to the TSPO response which was maximal at 7 days. In the SN, an acute microglial response was observed at 7 days, which was observed until 28 days after injection with TSPO-PET ligand [139]. In a 6-OHDA mouse model (2 μl of 5 mg/kg in the SN), Fricke et al. transduced cells in the subventricular zone with a lentivirus encoding for firefly luciferase to follow the migration of progenitor as a response to neurodegeneration induced neuroinflammation, which was imaged with [^18^F]DPA714 [145]. Finally, Nomura et al. showed that LPS-induced peripheral inflammation exacerbated the neuroinflammatory response to 6-OHDA. 6-OHDA lesioned animals treated with LPS showed higher expressions of pro-inflammatory cytokines at the site of the lesion, which was correlated with an increased TSPO-PET signal [146]. In conclusion, the 6-OHDA induces neuroinflammation-associated neurodegeneration, even though the neuroinflammatory reaction should rather be considered as acute, which is not representative of the slow chronic evolution of PD.

The lipophilic compound 1-methyl-4-phenyl-1,2,3,6-tetrahydropyridine (MPTP), after crossing the blood-brain barrier, is metabolized by astrocytes. The metabolite, MPP+, is transported into dopaminergic neurons through the DAT where it accumulates and causes toxicity through binding at the mitochondrial complex I. The first PET imaging study on this model was performed on the porcine brain by Cumming et al. who first identified the cerebral distribution and saturation parameters of TSPO binding sites (at that time still referred to as PBBS) by a quantitative [^3^H]PK1195 binding study. Subsequent in vivo *PET* imaging with [^11^C]-R-PK11195 was not successful in Landrace pigs despite the high presence of TSPO-binding sites; in Göttingen minipigs, a displaceable signal was detected, which tended to increase 2 weeks after MPTP treatment. Overall, they concluded that [^11^C]-R-PK11195 has a too low specificity to detect TSPO-binding sites in the porcine brain [147]. Chen et al. evaluated in the NHP, the time course of dopaminergic neuronal changes and neuroinflammation after chronic MPTP intoxication. In this parkinsonian model, they reported a transient increase in TSPO-PET uptake, measured by [^11^C]-R-PK11195, which coincided with a decrease in striatal vesicular monoamine transporter type 2 (VMAT2) binding before any decrease in DAT binding was observed [138]. Another study, also in the NHP MPTP model, reported on the neuroinflammatory response in relation to gender, neuroinflammatory cytokines and gut microbiota. It was shown that early and robust [^18^F]FEPPA PET signals coincided with earlier and more severe parkinsonism, which were especially seen in male compared to female NHP [148]. MPTP-induced neuroinflammation has further been applied in TREM2-deficient mice, using [^11^C]-R-PK11195, to demonstrate a central role of TREM2 in the regulation of microglial response to acute neurotoxic insults. Additionally, these data suggested a potential modulatory role of TSPO in response to immune system deficit [149].

Proteasomal inhibitors, such as lactacystin, cause dose-dependent dopaminergic neurodegeneration and are an alternative approach to model PD. In this context, a recently developed minipig parkinsonian model [150] has been evaluated in a multitracer PET study to investigate the longitudinal effect of chronic intracerebroventricular (ICV) exposure on monoaminergic projections and neuroinflammation, as evaluated with [^11^C]-PK11195 [151]. The results of this promising study showed that this model might better reflect early mechanisms in Parkinson’s disease pathology.

Transgenic or viral vector-induced models allow the following-up of neuroinflammation alongside progressive neurodegeneration. Rodriguez and colleagues characterized, in a humanized α-synuclein associated adenoviral vector-induced (AAV-hα-syn) rat model, the neuroinflammatory response in the SN using a longitudinal follow-up study. They showed that increased TSPO-PET binding measured with [^18^F]DPA-714 coincided with an increased number of Iba-1^+^ cells in the SN, while astrocytic activation occurred only at a much later stage. A significant correlation was shown between [^18^F]DPA-714 BP_ND_ and the number of Iba-1^+^ cells, but not with GFAP^+^ cells. Interestingly, the microglial reactivity preceded the reduction of TH^+^ cells, and over time, [^18^F]DPA-714 BP_ND_ was inversely correlated to the number of TH^+^ cells [136]. Surprisingly, in this model, the P2X7R tracer [^11^C]JNJ717 was not able to detect any microglial response [139, 152]. Unfortunately, no immunohistochemistry data identifying the glial cell type expressing TSPO has been reported. Finally, AAV-induced A53T overexpression in the SN of the minipig brain induced a significantly higher TSPO-PET uptake in the basal ganglia and cortical areas, as shown with [^11^C]-R-PK11195; however, neuroinflammation occurred without any detectable dopaminergic neuronal loss or behavioural deficit [153]. It was suggested that multiple injection sites or different vectors should be tested to improve the transduction of dopaminergic nigral neurons.

Finally, the role of LRRK2 in the immune system underlies one of many hypotheses why LRRK2 p.GS20195S mutation is one of the most common risk factors for PD. As peripheral administered LPS induces a neuroinflammatory response that eventually can lead to specific loss of dopaminergic neurons [15], LPS is an appealing tool to assess the association of peripheral-induced neuroinflammation in the progression of PD. In this context, a longitudinal [^11^C]PBR28 TSPO-PET study was performed on rats carrying the LRRK2 p.GS20195S mutation and non-transgenic littermates treated peripherally with LPS (3mg/kg, i.p.) or vehicle. This study revealed a significant increased TSPO-PET signal in transgenic rats 10 months after systemic LPS challenge compared to saline-treated non-transgenic littermates [22]. Interestingly, these authors calculated age-centred SUV by subtracting the mean SUV of all saline-treated animals from each individual rat’s SUV to account for the effect of age on neuroinflammation. This was based on previous work of the same group [154], where they reported on the sensibility of [^11^C]PBR28 TSPO-PET to ageing, which might be related to increased microglial activity in the ageing brain. In addition, in this study, the authors validated that the SUV measure is highly correlated with and less variable than the V_T_ quantification parameter.

### Huntington disease

Huntington’s disease (HD) is caused by a polymorphic trinucleotide CAG repeat expansion in the HTT gene that encodes the polyglutamine (polyQ) repeat in the N-terminal region of Huntingtin (Htt). These expansion repeats induce neuropathological hallmarks including a substantial accumulation of the Htt aggregates in the cortex and striatum [156]. These Htt-aggregated fragments set in motion a complicated cascade of both damaging, compensatory molecular processes and neuroinflammation, which is an important early pathological process of the disease. Activated microglia have been detected in brains from presymptomatic HD carriers [157] to post-mortem HD patients [158] together with elevated inflammatory cytokines in both the CNS and plasma from HD patients [159].

Finding from animal models have an important contribution to elucidate pathways that are disrupted and have provided insights into the pathogenesis of this disease, enabling the development of therapeutic strategies [160]. As no spontaneous occurring animal models of HD exists, this pathology is modelled by toxins and viral vectors or through the use of transgenic animals.

The administration of either excitotoxic agents – such as quinolinic acid (QA) – or mitochondrial toxins, such as 3-nitropropionic acid (3NP), can replicate some elements of the disease in both rodents [161, 162] and non-human primates (NHP) [163–165]. QA is an endogenous NMDA receptor agonist with excitotoxic properties. The excessive activation of NMDA receptors leads to a massive increase of calcium influx in neurons that involves the production and release of free radicals (reactive oxygen and nitrogen species), which can trigger cell oxidative damage leading to neuronal death. This model has been characterized by severe and fast (within 48 h) neuronal degeneration (GABAergic medium spiny neurons) as shown by the loss of NeuN staining [166]. Factors released during this degenerative process rapidly induce a pro-inflammatory environment leading to the activation of surrounding microglial cells and astrocytes [167]. Intracerebral injection of QA results in a reproducible lesion that –when injected in the striatum – reproduces some biochemical, behavioural and pathologic features of HD in rodents and non-human primates [168, 169, 167, 164]. In the 3NP (3-nitropropionic acid) model, Ramachandran et al. [170] have reported increased mRNA expression of pro-inflammatory markers and of GFAP compared to controls 14 days after toxin administration.

TSPO-PET imaging in HD models has mainly been reported in the QA excitotoxic model since the localized, transient neuroinflammatory lesion facilitates the short-, to mid- and to long-term evaluation of TSPO-PET/SPECT radiotracers. A summary of the TSPO-PET imaging studies in HD is provided in Table 6. In all TSPO-PET/SPECT imaging studies reported, QA was unilaterally injected in the striatum, using the contralateral side as an internal control. All excitotoxic QA models were induced in rodents, except for one study that reported TSPO imaging of [^18^F]-DPA-714 in non-human primates [171]. As such, the QA model has been used either to characterize the properties of new TSPO-PET/SPECT radiotracers ([^125^I]CLINDE [168, 172], [^18^F]DPA-714 [171], [^3^H]-PK11195 [173]) or to evaluate therapeutics or TSPO ligands as neuroprotective agents [174, 175]. Furthermore, short- to long-term NI changes post QA lesion were explored in transversal and longitudinal TSPO-PET imaging studies to correlate TSPO expression with glial reactivity in this model [172, 171, 176]. Overall, these studies reported a concomitant increase in TSPO radiotracer binding together with an increase in IHC inflammatory markers in the lesion. Arlicot et al. evaluated the properties of the [^125^I]CLINDE tracer at 6 days post QA injected at different doses [168]: microglial activation in the ipsilateral striatum was clearly observed using immunohistochemical (IHC) staining (OX-42 antibody), and authors evidenced a positive relationship between the intensity of IHC OX-42 staining and the dose of injected QA. Longitudinal TSPO-PET studies up to several months post-injection have also compared the time course of neuroinflammation with neurodegeneration, evaluating additionally the relative contribution of activated microglia and activated astrocytes in TSPO overexpression. Overall, longitudinal studies described a temporal transitory neuronal loss and reactive gliosis in the lesioned striatum. In Arlicot et al., TSPO expression was elevated from day 4 to 30 after QA administration with a maximal [^125^I]CLINDE binding at 4, 7 and 14 days post-injection followed by a decline from day 30 down to day 90 [172]. In Lavisse et al., the level of TSPO immunoreactivity markedly increased from day 7 and was maximal between day 21 and day 40 and then decreased in intensity until day 91 [171]. The [^18^F]-DPA-714 total distribution volume increased initially in the restricted centre of the injection site from day 7 and progressively encompassed a larger area from day 7 to day 21 and remained visible on day 49. The same spreading observation was reported by Moresco et al. with a parallel increase of [^11^C]-R-PK11195 binding and microglial activation (and macrophage infiltration) markers as revealed by OX-42 staining, in both striatal and extrastriatal areas. These increases were maximum 7 days after QA injection, but OX-42 staining disappeared at later time points (30 and 60 days) while [^11^C]-R-PK11195 binding was still increased although in the restricted lesioned area. This observation led to hypothesize the presence of reactive astrocytes as [^11^C]-R-PK11195 presumably binds also to this cell type [177].

**Table 6:** Overview of the preclinical TSPO-PET/SPECT imaging studies in Huntington’s disease models

PET/SPECT tracer	Rational	HD model and species	Imaging time points	Main imaging findings	Additional readouts	Ref.
[^125^I]CLINDE	Characterization and validation of the tracer at different stages of excitotoxic lesion	QA (*); 75, 150, 300 nmol in male Wistar rats	6 days post-injection	• Specificity confirmed with PK1195 blocking studies (-82% decrease). • Significant [^125^I]CLINDE uptake increase in lesioned compared to intact side	Autoradiographic analysis (including blocking) and IHC confirmed in vivo results	[168]
[^125^I]CLINDE	Investigation of the spatial and temporal density of TSPO after excitotoxic lesion	QA (*) 150 nmol in male Wistar rats	1, 7, 14, 60, and 90 days post-injection	• Uptake sign increased in the lesioned striatum from 1 to 60 days • Uptake increase until day 4 and plateau at 4–30 days. • Progressive decrease at 30–90 days	Autoradiography of TSPO expression corresponded to the temporal profile of both microglial activation and astrogliosis. Maximal astrocytic response at 7–14 days and at 14 days for microglia	[172]
[^11^C]-R-PK11195	Combination of in vivo, ex vivo and IHC approaches to analyse both short- and long-term changes in the QA model	QA (*) 210 nmol in Wistar rats	8, 30 and 60 days post-injection 30 and 60 days post-injection	At 8, 30 and 60 days, [^11^C]-R-PK11195 binding values are found to be 3.4, 3.0 and 2.8 times higher than those of the control	Reduction of both A2A and dopamine D2 receptors together with an augmentation of microglial activation/macrophage infiltration both in the lesioned striatum and, to some degree, also in extrastriatal areas	[176]
[^18^F]DPA-714	Pharmacological Characterization of the tracer and evaluation of the cellular contribution to the PET signal	QA (*) 180 nmol in cynomolgus NHP	7, 21, 40 90 days post-injection	• VT : +17%, +54%, +157% and +39% higher than baseline on days 7, 14, 21 and 91. • Decrease of uptake (-73%) in the lesioned striatum after blockage with PK11195	IHC demonstrated progressive microglial activation from day 2 followed by delayed astrocytic reaction reaching maximum between 7 and 14 days. High correlation with in vivo results (*r*^2^ = 0.98)	[171]
[^18^F]PBR06	Evaluation of the feasibility to detect activated microglia in these models	R6/2 , BACHD transgenic mice	Early, mid- and advanced stages	In R6/2 mice (advanced stage): • [^18^F]PBR06 accumulation in each ROI significantly higher by 25–30% compared with WTs • Reduction of 39–45% of uptake after blocking with PK11195 • Elevated [^18^F]PBR06 uptake in BACHD mice (early stage) compared with WTs in each ROI	TSPO expression correlated to microglial activation (increased IBA-1) and increased striatal levels of pro-inflammatory cytokines (IL-6 and TNFa)	[187]

In some of these longitudinal studies, the astroglial and microglial activation, in parallel to neuronal loss, was characterized at several time points from 1 to 60–90 days post-QA injection, by double or triple immunostaining with GFAP, OX-42, Iba1 or CD68 and TSPO antibody. Moresco et al. used complementary autoradiography and confocal laser-scanning microscopy techniques to explore striatal and extrastriatal changes after QA injection with a finer analysis of cellular and subcellular events triggered by QA injection [176]. The time course of [^125^I]-CLINDE binding matched with the temporal profile of both microglial and astrocyte reaction in Arlicot et al. study [172]: increased staining of GFAP-immunoreactive astrocytes was maximal at days 7 and 14 while microglial IHC marker peaked at day 14. They both then significantly decreased at days 60 and 90. Since [^125^I]-CLINDE binding followed the same temporal profile and co-localized in autoradiographic slices, authors concluded that both reactive astrocytes and microglia contributed to TSPO expression and signal. These observations were not in agreement with the results observed by Ryu and colleagues who reported that TSPO was primarily expressed in immunoreactive microglia and weakly in GFAP immunoreactive astrocytes, 24 h after QA injection [175]. This discrepancy could be partly explained by the different dose of injected QA used in these two studies (150 and 60 nmol in Arlicot et al. and Ryu et al., respectively) and the early time of imaging as astrogliosis has been reported to occur at later stages after lesion. Moreover, intrastriatal QA lesion in the NHP has shown to induce progressive microglial activation from day 2 (Iba-1 labelling) with a delayed astrocytic reaction reaching maximal expression at 7 and 14 days [171]. The activated microglia were detected in the core of the lesion area whereas cell bodies of reactive astrocytes were organized as a perilesional rim with long and straight processes entering the lesion core from the periphery, as previously reported in rats [178]. Triple immunostaining studies (TSPO, GFAP and CD68 antibodies) provided further evidence that the [^18^F]DPA-714 PET signal primarily originated from activated microglia in this model (TSPO and CD68 staining co-localization) although it had previously been noted that this TSPO radiotracer binds to reactive astrocytes in a model of selective astrocytes reactivity [179]. Indeed, reactive astrocytes are known to be molecularly and functionally heterogeneous and their molecular profile can depend on the induced-disease context, the disease stage or the considered brain region [180].

Neurotoxin models were developed before the discovery of the Htt genetic mutation. The identification of the genetic mutation in HD led to the generation of a variety of animal models that express different forms of mutant huntingtin (expression of either full-length or N-terminal fragments of mutant Htt) showing different pathological spectra of the disease [181]. For example, the R6/2 mouse model carrying an N-terminal exon 1 fragment of the disease-causing human HTT gene displays physiological and behavioural phenotypes including progressive weight loss, shortened life span, progressive motor dysfunction and cognitive decline [182, 183]. A wide range of gene dysregulations has been reported in various brain regions of R6/2 mice including the expression of multiple inflammation- and stress-related genes as well as genes related to neurodegeneration [184]. In the brain of the R6/2 mouse model, significantly elevated pro-inflammatory cytokines were detected such as interleukin 6 (IL-6) and tumour necrosis factor alpha (TNFα) [185]. At early disease stages, microglia have been shown to be abnormal through ferritin accumulation and Iba1 immunostaining, and these abnormalities correlated with disease severity [186]. However, to the best of our knowledge, only one TSPO imaging study in transgenic models has been reported yet. In two HD mouse models, Simmons et al. assessed the feasibility of utilizing TSPO-PET imaging with the [^18^F]-PBR06 ligand to detect activated gliosis [187]. [^18^F]-PBR06 revealed microglial activation at a late disease stage in R6/2 mice, and at early to mid-stage in symptomatic BACHD mice. The [^18^F]-PBR06 TSPO-PET signal was correlated with increased Iba-1 and TSPO IHC staining in both models, and correlation was particularly strong in the striatum, cortex and hippocampus while GFAP levels did not correlate with [^18^F]-PBR06 uptake. These results indicated that TSPO seems to be predominately expressed on activated microglia in the brain in these transgenic models.

(*), unilaterally injected in the striatum; *IHC*, immunohistochemistry; *WT*, wild type

#### Models of multiple sclerosis

The PubMed search used for this paragraph was “(omega* OR TSPO OR PBR OR PK11195) AND (PET OR positron) AND (brain OR cereb*) AND (multiple AND sclerosis) AND (rat OR mouse OR mice OR animal* OR experimental OR pig OR gerbil OR rabbit OR guinea OR model OR dog OR cat) AND (English[ lang])”; it returned 54 references, including 23 reviews; once Molecular Imaging and Contrast Agent Database entries, clinical studies and reviews were excluded, 11 preclinical studies were kept for this review. All these studies are summarized in Table 7.

**Table 7:** Overview of the preclinical TSPO imaging studies in models of multiple sclerosis

PET tracer *	Rational	MS model and species	Imaging time points	Main imaging findings	Additional readouts	Ref.
[^11^C]-R-PK11195	Advance in vivo imaging methodology for studying microglial activation and therapeutic response to minocycline after WM lesion	Zymosan A stereotaxic injection in the CC of female SD rats (250±19g)	7 days post-injection	• **Increased [**^**11**^**C]-R-PK11195 uptake in the zymosan-injected WM** • **46% reduction in [**^**11**^**C]-R-PK11195 uptake in the minocycline-treated group**	[^3^H]-R-PK11195 ARG confirmed the presence of NI in the zymosan-injected WM	[195]
[^18^F]PBR111	Investigate NI at different phases of EAE	Subcutaneous injection of PLP_139-151_ peptide in female SJL/J mice	Baseline and 6, 13, 20, 27, 35 and 41 days post-immunization	• **Peak increase (+135±20%) in [**^**18**^**F]PBR111 uptake in all brain ROIs after the 1st episode** • **Return to baseline during the 2nd episode** • **Significant increase during the 3rd episode**	IHC for TSPO and F4/80 and CD11b confirmed the increase in TSPO expression observed by PET to be microglial, GFAP+ cells were negative for TSPO	[188]
[^11^C]-R-PK11195 [^11^C]MeDAS (myelin) [200] [^18^F]FDG	Assess the feasibility of in vivo monitoring of MS-specific disease processes with PET	Demyelination by stereotactic injection of 1% lysolecithin in the CC and striatum of male SD rats (8–10 weeks old)	[^18^F]FDG and [^11^C]-R-PK11195 imaging at 3 days and 1 and 4 weeks and [^11^C]MeDAS PET at 1 and 4 weeks after injection	• No change in [^18^F]FDG uptake • **+84% and +37% in [**^**11**^**C]-R-PK11195 SUV in the lesion and ipsilateral hemisphere at 3 days and 1 week, returning to baseline at 4 weeks** • Decrease (-7%) in [^11^C]MeDAS SUVr	• Iba1 IHC confirmed the increase in NI in the CC and striatum 3 days and 1 week post-injection • [^11^C]MeDAS ARG showed a 57% decrease in myelin binding in ipsilateral CC • Myelin IHC confirmed demyelination at 3 days and 1 week and partial remyelination at 4 weeks	[198]
[^11^C]-R-PK11195 [^18^F]FDG [^18^F]FSPG (system xc^-^) [103]	Better understanding of system xc^-^ in NI in MS	Subcutaneous injection of MBP in male Lewis rats (8 weeks old, 200–220 g)	Baseline and at 7, 14, 21 and 28 days after EAE induction	• No significant changes in FDG • **Significant increase in [**^**11**^**C]-R-PK11195 uptake in the cerebellum and cervical and lumbar spinal cord at 14 days** • Significant increase in [^18^F]FSPG uptake in the lumbar spinal cord at 14 days, normalized by administration of liposome-encapsulated clodronate	• Liposome-encapsulated clodronate treatment tends to worsen neurological score but reduced IHC Iba1 staining • Suggest that microglia are the source of system xc^-^ in NI	[199]
[^18^F]VC701	Evaluate NI in a mouse EAE using TSPO-PET with [^18^F]VC701	Subcutaneous injection of MOG_35-55_/CFA and pertussis toxin in female C57BL/6J mice (8–12 weeks old)	14 days after EAE induction	• **Increased [**^**18**^**F]VC701 SUVr in the cortex, cerebellum, striatum, hippocampus, cervical enlargement and thoracic and lumbar spinal cord**	• PET data confirmed by ex vivo biodistribution of [^18^F]VC701 • Iba1 IHC confirmed the presence of microglia/infiltrated macrophages in the same brain ROIs as PET • EAE lesion visible on MRI but highly variable in volume	[189]
[^11^C]PBR28 [^18^F]FOL (FR-β) [201]	Investigate FR-β expression and evaluate its potential as an in vivo imaging target in comparison of TSPO	Intrastriatal injection of heat-killed BCG followed by intradermal injection of *Mycobacterium tuberculosis*- H37Ra in male Lewis rats (3–4 months old, 235±9 g)	14 and 90 days after EAE induction	• **Increase in [**^**11**^**C]PBR28 SUVr 14 and 90 days after EAE induction** • Increase in [^18^F]FOL SUVr, similar to [^11^C]PBR28 SUVr, at 14 days • **[**^**18**^**F]FOL SUVr higher than [**^**11**^**C]PBR28 SUVr at 90 days**	• ARG for [^11^C]PBR28 and [^18^F]FOL confirmed the PET data • IHC confirmed the presence of FR-β, CD68, MRC1 and iNOS staining in the same ROIs as PET, especially at 90 days	[202]
[^18^F]GE-180	Feasibility study of using TSPO-PET to detect NI in EAE and determine which cell types express TSPO	CPZ-induced EAE and/or MOG_35-55_ immunization in C57BL/6 mice and hGFAP/EGFP (astrocyte), CX3CR1^+^/eGFP/CCR2^+^/RFP (monocyte-derived macrophages) and eGFP-expressing microglial transgenic mice	5 weeks after EAE induction	•**Increase of [**^**18**^**F]-GE180 uptake in CPZ-treated mice (+36 to +65% vs control mice)**	• IHC analysis revealed that TSPO mostly colocalize with microglia following CPZ • The combination CPZ and/or MOG_35-55_ induces the recruitment of TSPO+ monocytes	[190]
[^18^F]GE-180	Test effect of anti-VLA-4 treatment in EAE	Intrastriatal injection of heat-killed BCG followed by intradermal injection of *Mycobacterium tuberculosis* in male Lewis rats	30, 44, 65, 86 and 142 after EAE induction	• **Declining trend (*p*** **= 0.067) in [**^**18**^**F]GE-180-binding after 2 weeks of anti-VLA-4 mAb-treatment vs controls** • **After 31-days of anti-VLA-4 mAb treatment, cessation of treatment increased [**^**18**^**F]GE-180 binding vs control group** • **No difference between groups in TSPO binding by day 142**	IHC confirmed the presence of Iba1^+^ cells in the lesion	[191]
[^18^F]DPA-714	Investigate the temporal profile of NI in relation to MRI in EAE	CPZ-induced EAE in female C57Bl6 mice (8 weeks old, 19.8±1.5 g)	4 and 5–6 weeks after EAE induction	• **[**^**18**^**F]DPA-714 increased in EAE mice at 4 weeks and declined at 6 weeks in the CC, hippocampus and thalamus** • Increased T2 values in the CC of CPZ compared to control at 3 and 5 weeks • Partial recovery of T2 values between weeks 3 and 5	• Ex vivo [^18^F]DPA-714 ARG confirmed the PET data • IHC: during demyelination (week 3), TSPO^+^ cells are microglia; during remyelination, TSPO^+^ cells are astrocytes • IHC confirmed remyelination at week 6	[193]
[^18^F]GE-180	Test MS treatment laquinimod in EAE model	CPZ-induced EAE ± MOG_35-55_ immunization in female C57Bl6 mice (8 weeks old)	5 weeks after EAE induction	• **Laquinimod treatment returned EAE-induced increased [**^**18**^**F]GE-180 uptake to control values**	IHC measurements confirmed reduced NI and decrease of ND markers	[192]
[^18^F]DPA-714	Feasibility study of using TSPO-PET and SPIO-MRI to detect NI in EAE and determine which cell types express TSPO	Immunization with PLP_139-151_ of female SJL/J mice (6 weeks old)	11 to 14 days post-immunization	• **[**^**18**^**F]DPA-714 SUVR and SPIO-volume values were significantly increased in EAE compared with the controls in the hippocampus, thalamus, cerebellum and brainstem** • Increased SPIO-Vol only in the caudate/putamen	TSPO/Iba1 and F4/80/Prussian blue IHC staining suggests that microglia and macrophages are the source of [^18^F]DPA-714 and SPIO signal	[194]

*TSPO-PET tracers and associated results are in bold.

Abbreviations: *[*^*18*^*F]FDG*, fluorodeoxyglucose; *[*^*18*^*F]FOL*, [^18^F]fluoride-labelled 1,4,7-triazacyclononane-1,4,7-triacetic acid conjugated folate; *[*^*18*^*F]FSPG*, (4S)-4-(3-18F-fluoropropyl)-L-glutamate; *ARG*, autoradiography; *BCG*, Bacillus Calmette-Guérin; *CC*, corpus callosum; *CPZ*, cuprizone; *EAE*, experimental autoimmune encephalomyelitis; *FR-β*, folate receptor-β; *IHC*, immunohistochemistry; *iNOS*, inducible nitric oxide synthase; *MBP*, myelin basic protein; *MeDAS*, N-methyl-4,4′-diaminostilbene; *MRC1*, mannose receptor C-type 1; *ND*, neurodegeneration; *NI*, neuroinflammation; *SD*, Sprague Dawley; *SPIO*, superparamagnetic iron oxide particles; *SUV*, standard uptake value; *SUVr*, standard uptake value ratio; *VLA-4*, very late antigen-4 integrin; *WM*, white matter; *xc*^*-*^: cystine-glutamate antiporter system

Various studies investigated the neuroinflammatory profile of different models of MS (EAE or WM lesion) using [^18^F]PBR111 [188], [^18^F]VC701 [189], [^18^F]GE-180 [190–192] or [^18^F]DPA-714 [193, 194] PET tracers. Overall, TSPO imaging studies in MS models consistently reported a moderate to large increase in TSPO tracer uptake in the affected brain regions. Using an interesting model of relapsing experimental autoimmune encephalomyelitis (EAE) in mice, Mattner et al. [188] demonstrated a high increase (+135%) in [^18^F]PBR111 uptake during the 1st episode before the signal returned to baseline during the 2nd episode and bounced back up during the 3rd episode, demonstrating the interest of using TSPO imaging to monitor different stages of the disease in the preclinical model but with also important implication for translational application. The in vivo PET data were confirmed by IHC, demonstrating that TSPO expression was limited to microglial cells while astrocytes were TSPO negative. Additionally, Nack et al. [190] reported that TSPO^+^ cells were microglia following cuprizone treatment and that supplementing Cuprizone with MOG_35-55_ peptide immunization drove the recruitment of TSPO^+^ monocytes in the lesion contributing to the overall PET signal on top of resident activated microglia; these results are in agreement with a more recent study with [^18^F]DPA714 [194]. Interestingly, Zinnhardt et al. [193] showed using [^18^F]DPA714 that during demyelination TSPO^+^ cells consisted only of microglia, while during remyelination, astrocytes also became TSPO^+^. Using [^11^C]-R-PK11195 TSPO-PET as a potential readout for therapeutic intervention, Converse et al. [195] aimed to evaluate the response to minocycline, an antibiotic that also represses microglial activation, in the zymosan-induced WM lesion in rats. They demonstrated an increase in [^11^C]-R-PK11195 uptake in the WM lesion that was significantly reduced by minocycline. More recently, Vainio et al. [191] investigated the response to an anti-VLA4 (very late antigen-4 integrin) antibody treatment in an EAE model in rats and demonstrated only a trend to increase in [^18^F]GE-180 uptake following 2 weeks of treatment but interestingly a rebound in neuroinflammation at the cessation of the treatment at 31 days, suggesting a dampening effect of the treatment on the pathophysiological processes. Similarly, Nedelcu et al. [192] investigated the effect of laquinimod in a mouse model of MS also using [^18^F]GE-180. These authors demonstrated that laquinimod was able to reverse neuroinflammation to baseline levels confirming the potential of this treatment in MS as previously described [196, 197].

Some studies used a multitracer approach to investigate different parameters simultaneously in MS models. In a model of demyelination in rats, de Paula Faria et al. [198] used [^11^C]-R-PK11195 for TSPO expression, [^11^C]MeDAS for myelin level and [^18^F]FDG for metabolism. While they showed no change in [^18^F]FDG uptake, they observed a significant increase (+37 to +84%) in [^11^C]-R-PK11195 uptake in the lesion where myelin binding of [^11^C]MeDAS was significantly decreased within a week. Similarly, Martin et al. [199] also found no change in [^18^F]FDG uptake in an EAE model in rats, while TSPO ([^11^C]-R-PK11195) was significantly increased in the cerebellum and spinal cord, and cystine-glutamate antiporter system (system xc^-^) ([^18^F]FSPG) was increased in the spinal cord 14 days post-EAE induction. As for the study in stroke [97], this raises the prospect of system xc^-^ being involved in neuroinflammatory processes in MS and being a potential therapeutic target. Finally, and using [^18^F]FOL to target folate receptor-β (FR-β) that is expressed on activated macrophages, Elo et al. investigated the use of FR-β as a potential biomarker for neuroinflammation imaging in MS in comparison with [^11^C]PBR28 PET. Fourteen days post-EAE induction, both [^11^C]PBR28 and [^18^F]FOL were similarly increased; however, at 90 days post-EAE induction, [^18^F]FOL uptake was higher than [^11^C]PBR28 uptake. Immunohistochemistry revealed that FR-β was expressed by both microglia and macrophages in EAE lesion; however, the different uptake of both tracers at 90 days suggests that [^18^F]FOL/FR-β imaging may be complementary of TSPO imaging in MS.

Overall, in preclinical models of MS, TSPO expression is robustly and consistently detected as it is the case in MS patients. In this context, TSPO imaging has a great potential as a readout for therapeutic response (Table 7).

## General conclusion

Taken altogether, TSPO-PET imaging is highly valuable in assessing the time course, anatomical brain localization of neuroinflammation and response to treatment in a range of disease models from acute, subacute to chronic conditions. We observed that the TSPO-PET ligands that have been developed over the last 15 years to overcome the shortcomings of [^11^C]-R-PK11195 have been applied in a large variety of preclinical studies. To the best of our knowledge, the differential affinity of these ligands for the human TSPO polymorphisms has still not been an issue in preclinical research nor in larger animals. Recently, newer ligands, which are not sensitive to these polymorphisms, are integrating the field. Future studies will define if these ligands present an overall gain in preclinical research over [^11^C]-R-PK11195 in terms of signal-to-noise ratio. Clinical studies will then have to determine if this gain is translated into clinical imaging and if they are truly insensitive to the TSPO polymorphism in human.

Overall, we can conclude that TSPO-PET imaging is a powerful tool to evaluate the neuroinflammatory response in models with strong and localized effects, such as intracerebral administered toxic lesion models (LPS, QA, 6-OHDA) and stroke models. The main advantage of these models is the reproducibility and predictiveness of the size, localization and the time window of the neuroinflammatory response as well as the known cell origin of the TSPO expression. These parameters reduce considerably the inter-animal variability and simplify the experimental design. However, the major drawback of these models of acute neuroinflammation is the limited translational generalizability for neurological disorders with a systemic or chronic pathogenesis. This limitation highlights a strength of acute LPS administration, which can be given systematically, to model sickness behaviour and peripheral increase of inflammatory cytokines on the central neuroinflammatory response. However, dramatic species differences in LPS sensitivity are a key for translational consideration. On the other hand, viral vector-induced models or transgenic animal models of neurodegenerative diseases have the merit to better reflect the clinical pathology because they are slowly progressive, even though neuronal loss is often moderated within the time window of the experimental design. However, they also generate higher intra-animal variability and are more complex to handle in their experimental design. Additionally, the neuroinflammatory response in these models is often more modest and less well characterized but actually reproduces fairly well the amplitude and characteristics of the neuroinflammatory response observed in a clinical scenario. In animal models with a low and localized neuroimmune response, the use of ex vivo or in vitro autoradiography compensates for the limited resolution and sensitivity of in vivo PET imaging. A more general limitation is the use of SUV as a pseudo-quantitative measurement to overcome the complexities of blood sampling in small animal models. However, SUV is rarely compared with gold standard measurements of *V*_T_ and/or verify the free blood fraction of the ligand. When this measure is not homogeneous between experimental groups, an important bias may be introduced when using pseudo-quantitative parameters. This supports the development and use of animal models in larger species, such as non-human primates and minipigs, to circumvent some of the difficulties encountered when using rodents (small brain and limited possibilities of arterial blood sampling). A summary of the strengths and weaknesses of preclinical models described in this review is provided in Table 8.

**Table 8:** Strengths and weaknesses of preclinical models regarding TSPO-PET imaging

**Model**	**Pros**	**Cons**	
LPS	• Intracerebral administration provides a robust neuroinflammatory response. • Useful to model the impact of systemic inflammation on neurodegenerative and neuroinflammatory events	• Important species differences in neuroinflammatory response after peripheral LPS challenge • Differences in neuroinflammatory responses between batches of LPS	• For most models: small brain size of rodents, particularly mice, relative to the resolution of preclinical PET scanners. • *Need to implement more clinically relevant models in larger species*
Stroke	Robust, focal and well-defined time course of the neuroinflammatory response	Complexity and/or invasiveness of surgery (depending on model)	
	Moderate to good translational value depending on the stroke model		
AD	Amplitudes of increases in TSPO expression are similar in amplitude to what is observed in clinic	Transgenic models are only modelling the familial form of AD	
PD	Within the time window of the experimental design, neurodegeneration in viral vector-induced models is generally mild, alongside a modest and yet poorly characterized neuroinflammatory response	Acute neurodegenerative models present transient neuroinflammatory response which is not representative for a chronic aspect of clinical profile	
HD	Robust, focal, strong, rapid and well-defined time course of neuroinflammatory response (QA)	Poor to moderate translational value; invasiveness of surgery (QA)	
MS	Easy to induce, rapid neuroinflammatory response		
	Poor to moderate translational value		

Altogether, these TSPO-PET imaging studies demonstrated the implication and possible modulation of neuroinflammation by various systems in preclinical models and offered new insights into disease mechanisms and associated potential new therapeutic avenues. Additionally, they showed that these phenomena can be monitored in vivo longitudinally using preclinical PET alone (using one or several radiotracers) or in combination with other non-invasive imaging techniques. Importantly, these studies confirmed further the true potential of preclinical PET imaging and the still undeniable value of TSPO-PET to image neuroinflammation even though TSPO might not be seen as the ideal direct biomarker for neuroinflammation.

Interestingly, all these preclinical studies highlight the strengths and values of preclinical models in which it is possible to investigate the detailed cellular expression of TSPO (in microglia, macrophages, astrocytes and endothelial cells) as well as the meaning of such overexpression in the context of the cellular phenotype (pro- vs anti-inflammatory) and in relation with the immune response observed in these disease models, something that can be done only at the latest stage of disease in human samples.

There are many arguments that can be raised regarding the imperfection of TSPO as a biomarker of neuroinflammation since TSPO is not directly involved in the inflammatory responses as are a wide range of molecule such as cytokines, chemokines, P2X7 receptors, inflammasomes, etc. Nevertheless, until other biomarkers and associated radiotracers can fulfil the gap of TSPO shortfall, TSPO remains a tool of choice to investigate and understand neuroinflammation and response to anti-inflammatory treatment in animal models, and it still has an incredible value as a translational target for neuroinflammation imaging in clinic.
